# Evaluating the ESP32-S3 for Wi-Fi Penetration Testing Through the Development of Deauther32 and HackHeld32

**DOI:** 10.3390/s26113287

**Published:** 2026-05-22

**Authors:** Stefan Kremser, Kalman Graffi

**Affiliations:** 1 Faculty of Technology, Computer Science and Economy, Bingen Technical University of Applied Sciences, 55411 Bingen am Rhein, Germany; 2 Faculty of Design, Computer Science and Media, RheinMain University of Applied Sciences and Arts, 65195 Wiesbaden, Germany

**Keywords:** Wi-Fi security, penetration testing, ESP32-S3 microcontroller, IoT security, wireless network vulnerabilities, open-source security tools, frame injection and capture, portable hacking devices, firmware development, smart environment security

## Abstract

Wi-Fi security analysis and testing tools are vital to ensure the safety of wireless networks. Specialised hardware and software are needed to examine the underlying technology that connects our devices wirelessly. This article explores the feasibility of utilising the ESP32-S3 microcontroller as the basis for a low-cost, open-source, portable Wi-Fi penetration testing tool. By developing and evaluating the Deauther32 firmware, the project demonstrates key functionalities such as capturing and injecting frames to execute common Wi-Fi attacks, like beacon flooding and deauthentication. The developed HackHeld32 design complements the firmware by offering a compact and extendable handheld device, making the tool standalone and portable. These prototypes build upon previous work, the ESP8266 Deauther and the HackHeld Vega, by introducing significant improvements in functionality, usability, and hardware capabilities. This establishes a strong foundation for future development by demonstrating the potential of microcontroller-based solutions. These tools bridge the gap between accessibility for beginners and functionality for professionals by offering a cost-effective and portable solution for Wi-Fi security testing and beyond.

## 1. Introduction

**Figure 1:**
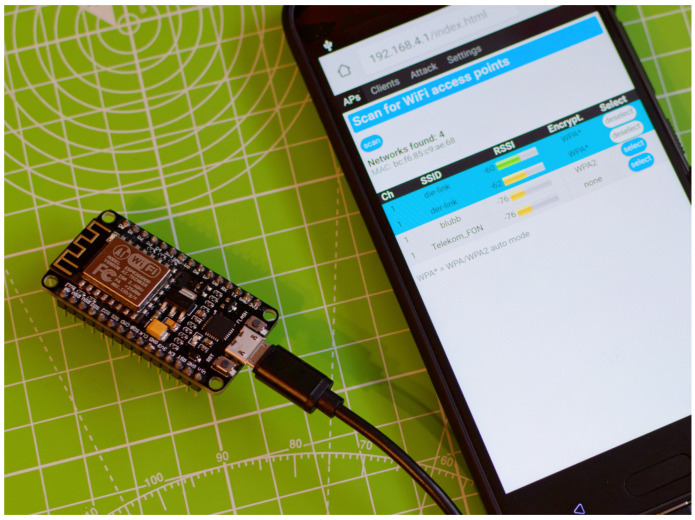
ESP8266 Deauther Controlled via Phone Web Interface.

Wi-Fi is used everywhere, from homes to businesses and public places. It is the technology that allows our devices to connect to the Internet wirelessly. Sending data over the air is convenient but comes with security risks. Someone nearby can intercept the data and potentially gain sensitive information. With the increasing number of automated devices that rely on Wi-Fi, disrupting connections becomes a serious threat, potentially causing malfunctions or interruptions in critical operations. In 2017, we developed a tool called the ESP8266 Deauther [1], making Wi-Fi security testing simple and affordable. As shown in Figure 1, it ran on a small microcontroller board and could be controlled from a phone. Its low price and ease of use made it popular among hobbyists and beginners. To offer this device in a compact handheld version, we designed the HackHeld Vega, turning the ESP8266 Deauther into a fully standalone tool. Since then, newer hardware, like the ESP32 family of microcontrollers, has been introduced, offering better performance and new features. We address the question of how to create a successor to the ESP8266 Deauther using new hardware to enhance performance and introduce new features while preserving the core values that made it so popular.

This question is at the heart of this article. It explores the new ESP32 microcontrollers and how to use them for Wi-Fi security testing, building on our earlier projects to develop a more capable and versatile tool.

### 1.1. Goals and Scope

This article aims to develop a Wi-Fi penetration testing tool based on one of Espressif’s (https://www.espressif.com/, accessed on 15 May 2026) ESP32 (https://www.espressif.com/en/products/socs, accessed on 15 May 2026) Wi-Fi microcontrollers. As the direct successor to the ESP8266, the ESP32 chips offer improved performance, additional memory, and new Wi-Fi capabilities. This makes them ideal candidates for building on the strengths of the ESP8266 Deauther while enabling new features. The focus of this article is on demonstrating the feasibility of using an ESP32 microcontroller for Wi-Fi hacking tasks by addressing technical constraints and adhering to the principles of simplicity and affordability. The ultimate aim is to lay the groundwork for a tool that is accessible to Do-It-Yourself (DIY) enthusiasts and beginners. This article reports only the functionality implemented in the current prototype; planned user-interface placeholders, such as the AP-detail-page controls for packet capture and Denial-of-Service (DoS) attacks, are treated as future work.

The scope of this work includes:

- Developing a hardware prototype to explore the advantages of newer hardware for portable Wi-Fi security testing.
- Implementing core functionalities essential for Wi-Fi hacking, such as capturing and sending Wi-Fi frames, to evaluate the hardware’s suitability.
- Creating a Deauther32 firmware prototype to test the feasibility of an ESP32-based successor to the ESP8266 Deauther.

### 1.2. Requirements

The goal of building upon the ESP8266 Deauther results in these requirements.

Hardware:

- Compact handheld form factor, not bigger than or comparable to a smartphone.
- Ability to run on battery power for over one hour.
- Hardware components that are widely available and simple to assemble, even for non-experts.
- Affordable price to ensure accessibility for hobbyists and DIY enthusiasts, ideally under 50 €.

Wi-Fi Functionality:

- Sending custom Wi-Fi frames (packet injection).
- Capturing Wi-Fi frames (monitor mode).
- Signal range sufficient to target nearby devices reliably, typically within the same room or 10 m.

Firmware:

- Intuitive User Interface (UI) that is easy to navigate.
- Simple and beginner-friendly installation and setup process.
- Support for custom “apps” to expand functionality.

### 1.3. Approach

The process started by selecting Espressif’s ESP32-S3 Wi-Fi microcontroller as the basis for the tool. Building on the HackHeld Vega, a “HackHeld32” hardware prototype was designed and assembled. Then, appropriate software tools and libraries were selected to develop the firmware, enabling the essential features needed for Wi-Fi hacking. Capturing and sending Wi-Fi frames were implemented, tested, and evaluated. Finally, a prototype of the “Deauther32” firmware was implemented, demonstrating its potential as a successor to the ESP8266 Deauther.

### 1.4. Key Contributions

This article presents notable advancements in developing inexpensive, open-source, DIY Wi-Fi hacking tools using microcontrollers, such as the ESP32-S3. The key contributions of this article are as follows:

- Integrated Handheld ESP32-S3 Platform: A compact, DIY-friendly handheld device was designed with a colour LCD, microSD card slot, and optional hardware extensions. Unlike existing ESP32-based tools, which are web- or command-line-based, HackHeld32 is a fully integrated, standalone handheld for Wi-Fi security testing, tackling critical usability gaps identified in prior work.
- GUI-Driven Deauther32 Firmware: A modern Graphical User Interface (GUI)-based firmware prototype was created as a practical successor to the ESP8266 Deauther, enabling true standalone operation without requiring a computer or smartphone. This represents a paradigm shift from existing ESP32 tools toward a handheld-first design.
- Low-Level Frame Capture and Injection Workarounds: Custom firmware techniques were developed to overcome the ESP32-S3’s hardware limitations in frame capture and injection, enabling capabilities that are not advertised by the manufacturer.
- Comprehensive Comparative Evaluation: Range tests, throughput analysis, and direct performance comparisons with professional tools (Wi-Fi Pineapple, Kali Linux) demonstrate that the ESP32-S3-based platform is viable for focused network analysis tasks despite hardware constraints—a finding that establishes practical viability.
- On-Device Firmware Management: A custom bootloader and app manager were implemented, enabling users to install and manage applications without a computer—a novel accessibility feature that differentiates this work and supports its accessibility argument.
- Affordability and Accessibility: The project balanced cost and functionality, making the HackHeld32 and Deauther32 accessible to hobbyists and DIY enthusiasts while ensuring expandability and ease of use.

These contributions establish the ESP32-S3 as a viable platform for low-cost Wi-Fi security tools and provide a solid foundation for further research and development in this domain.

## 2. Fundamentals

To recapitulate the basis for our approach, we describe the Wi-Fi standard and the elements that we utilise to implement our Wi-Fi penetration testing tool.

### 2.1. Wi-Fi Standard

Wi-Fi is the Wireless Local Area Network (WLAN) technology that enables wireless Internet access in homes, offices, and public spaces. Laptops, tablets, smartphones, and an increasing number of IoT devices, such as thermostats, lightbulbs, and refrigerators, rely on it. The IEEE 802.11 standard [2] defines how Wi-Fi works, and devices that claim to be Wi-Fi-supported must be certified to comply with it. Since its introduction in 1997, each major revision of the Wi-Fi standard has improved speed, range, and security. Wi-Fi operates across multiple frequency bands, most notably 2.4 GHz and 5 GHz. The hardware constraints of the ESP32-S3 caused us to focus on the 2.4 GHz band. Nevertheless, the development of a 5 GHz version remains feasible as newer ESP32 models are introduced.

Wi-Fi transmits data in frames, which encapsulate higher-layer packets and include Medium Access Control (MAC) addressing and a checksum for integrity. Three frame types serve distinct roles:

- Management frames handle network management tasks such as connecting, authenticating, and maintaining the connection.
- Control frames coordinate data transmissions, such as acknowledgment (ACK) and request-to-send (RTS) signals.
- Data frames carry the payload between devices, typically containing packets from higher-layer protocols.

An Access Point (AP) provides network access; a Station (STA) is any client device that connects to it. The AP continuously broadcasts beacon frames containing network information, including the Service Set Identifier (SSID) (network name), Basic Service Set Identifier (BSSID) (the AP’s MAC address), channel, and authentication method. Stations discover available networks by listening for these frames.

**Figure 2:**
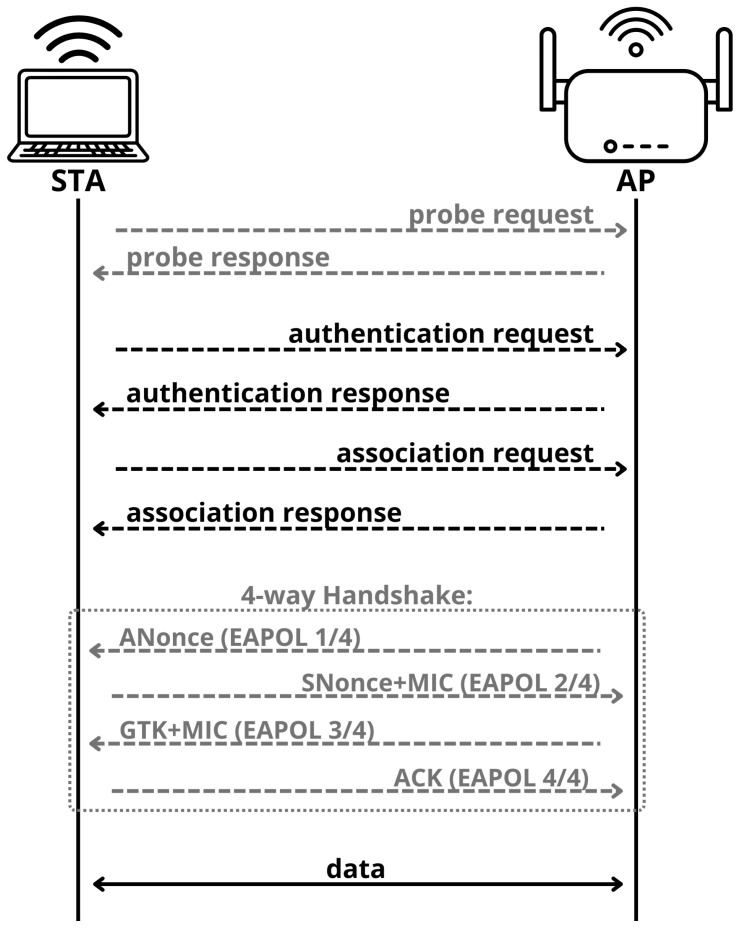
Wi-Fi Connection Process.

To connect, the STA authenticates with the AP by exchanging authentication frames and then sends an association request; the AP responds with an association response to complete the connection. Secure networks additionally perform a four-way handshake to derive encryption keys. Figure 2 shows the full connection sequence. Either party may terminate the connection at any time by sending deauthentication or disassociation frames.

**Figure 3:**
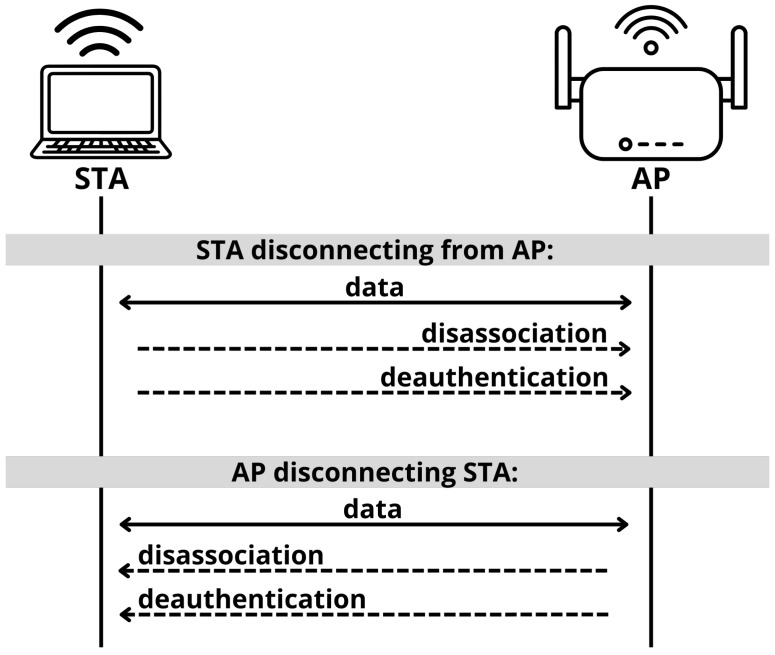
Wi-Fi Disconnection Process.

To disconnect, the STA disassociates and deauthenticates from the AP. Likewise, the AP can disassociate and deauthenticate the STA to force it to disconnect. This process is visualised in Figure 3. There are scenarios in which the STA disassociates without deauthenticating, allowing it to reconnect quickly without going through the entire reauthentication process. This is common when the STA is roaming between APs within the same network. Roaming refers to the process by which a STA switches between APs while maintaining its connection to the network. It is also possible to skip disassociation and deauthenticate only, terminating both the association and authentication processes immediately.

### 2.2. Wi-Fi Security and Vulnerabilities

Wi-Fi is a shared medium in which passive capture of nearby traffic, commonly referred to as sniffing, is possible. Security standards have evolved to protect this traffic: WPA2 uses AES encryption with a pre-shared key (PSK) and a four-way handshake to derive session keys, but the handshake can be captured and used for offline brute-force attacks against weak passwords. WPA3 addresses this with simultaneous authentication of equals (SAE) and mandates protected management frames (PMF) to prevent deauthentication and disassociation attacks. However, the effectiveness of PMF depends on implementation quality, and practical deauthentication attacks against PMF-protected WPA2/WPA3 deployments have been demonstrated [3].

PMF changes the practical impact of deauthentication testing: on networks where PMF is required, spoofed deauthentication frames are typically rejected, so the attack is no longer effective in the same way as on legacy networks. This makes the tool relevant for testing whether a network is truly enforcing PMF, especially in legacy WPA2 deployments and mixed transition environments as WPA3 adoption grows.

Beyond encryption weaknesses, several attack vectors remain relevant for Wi-Fi security testing [4]:

- Deauthentication and Disassociation Attacks: Attackers spoof management frames to disconnect devices from the network.
- Rogue APs and Evil Twin Attacks: A malicious AP is set up for Man-in-the-Middle (MiTM) attacks or phishing; an evil twin clones a legitimate AP to deceive users into connecting.
- MAC Address Spoofing: Networks that rely on MAC-based access control are vulnerable to attackers spoofing an authorised device’s address to gain access.

### 2.3. Hacking and Penetration Testing

Some devices implement protective measures against the attack vectors described above, while others remain vulnerable. Security testing of Wi-Fi networks and devices is therefore essential, particularly in settings such as businesses, public infrastructure, and industrial environments.

Ethical hackers perform Pentests under explicit authorisation to identify and remediate weaknesses before they can be exploited. The same technical methods can be used maliciously, so these tools are best understood as dual-use technologies.

Accordingly, this work frames hacking as a neutral technical term and focuses on lawful, controlled testing and education. In particular, deauthentication, beacon flooding, and any future features involving MiTM or phishing APs are discussed only as capabilities for authorised security testing in controlled environments.

### 2.4. Tools for Wi-Fi Hacking

Wi-Fi interfaces handle wireless communication for the host system, operating at the physical layer (Layer 1) and the data link layer (Layer 2) of the OSI model and managing tasks such as signal modulation, transmission, and medium access control (MAC) for seamless data exchange. For Wi-Fi hacking, access to Layer 2 is crucial. The network layer (Layer 3) and higher layers are fully accessible in most cases. However, Layer 2 is where Wi-Fi-specific functions occur. Access to it is needed to inject frames and capture Wi-Fi traffic. A visual representation of the OSI model is provided in Table 1.

Only with special software and hardware is it possible to utilise monitor mode, which allows capturing raw frames, and packet injection, which enables the transmission of custom frames. Technically, any Wi-Fi interface could be used for this purpose. However, in practice, only a few Wi-Fi chipsets are supported, and some require modified drivers to unlock their full potential. The firmware, drivers, or operating system often restrict hardware capabilities.

Software-defined radios (SDRs) offer an alternative, operating at Layer 1 and having the flexibility to capture and transmit raw signals. This freedom, unfortunately, also makes them impractical for Wi-Fi-specific tasks, as they lack built-in frame decoding and protocol compliance.

Therefore, tools like Aircrack-ng andWireshark are commonly used with supported Wi-Fi adapters on Linux distributions such as Kali Linux, a popular choice due to its extensive collection of preinstalled hacking tools. The time and hardware costs required to set up such aWi-Fi Pentesting system can be challenging for beginners, highlighting the need for more accessible and beginner-friendly solutions.

Details about the tools and hardware are discussed further in the following section.

## 3. Related Work

In this section, we discuss related projects that aim to hack Wi-Fi connections using Linux distributions, microcontrollers, and single-board computers.

### 3.1. Wi-Fi Hacking with Linux

Kali Linux (https://www.kali.org, accessed on 15 May 2026) is a distribution made explicitly for Pentesting, providing a large set of preinstalled hacking tools, including those for Wi-Fi hacking. The large community, open-source nature, and ability to run on most computers make Kali Linux a popular entry point into hacking.

**Figure 4:**
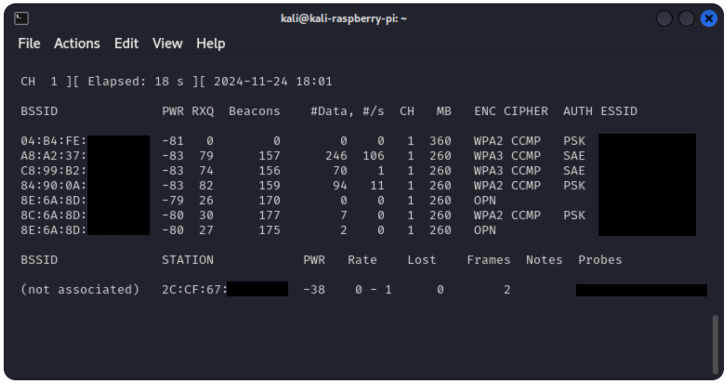
Aircrack-ng’s airmon-ng.

Aircrack-ng (https://www.aircrack-ng.org/, accessed on 15 May 2026) is one of these preinstalled Wi-Fi hacking tools. It is a suite of command-line tools for Wi-Fi scanning, capturing traffic, packet injection, and password cracking. In Figure 4, airmon-ng is displayed as an example of one of its tools. Due to Aircrack-ng’s versatility and reliability, it serves as the foundation for many other Wi-Fi hacking tools, such as AngryOxcide (https://github.com/Ragnt/AngryOxide, accessed on 15 May 2026).

“Is Your Wi-Fi Really Protected?” [5] explores the practical application of the Aircrack-ng suite for penetration testing on WPA2-protected networks, highlighting its effectiveness and raising awareness about securing Wi-Fi networks. Building on this, “Requirements for Secure Wireless Networks” [6] analyses the vulnerabilities of the WEP and WPA2 security standards. It demonstrates how Aircrack-ng is used to compromise these protocols. Together, these papers underscore the relevance of Aircrack-ng as a tool for testing Wi-Fi security.

Kali-Pi [7] is a Raspberry Pi 3-based ultra-portable pentesting device with a TFT touchscreen, battery, and Kali Linux for tasks like Nmap scanning, OpenVAS vuln assessment, and Metasploit exploitation. It prioritises general network pentesting portability over Wi-Fi-specific attacks, requiring more power-hungry SBC hardware and lacking a custom GUI, bootloader, or ESP32 optimisations.

While a modern computer provides the most capable setup, that solution has certain drawbacks. Setting up a separate environment for Pentesting is recommended, which can be time-consuming. Linux is typically used on larger machines that are less conveniently portable than our HackHeld device. Installing Linux may require dual-booting, using a virtual machine (VM), or enabling the Windows Subsystem for Linux (WSL). The command-line nature of most of these tools can present a steep learning curve for beginners. Additionally, a hacking-capable USB Wi-Fi adapter is required, often needing modified drivers or other workarounds.

The Nexmon framework [8] is one of these workarounds. It adds monitor mode and frame injection to Broadcom Wi-Fi chips. While powerful, it demands specific hardware and firmware expertise, emphasising the difficulty of using Wi-Fi interfaces for hacking.

These limitations highlight the motivation behind building a standalone Wi-Fi hacking device. A self-contained device eliminates the need for a specifically prepared computer, simplifies the workflow, and provides greater portability. With the focus on affordability and ease of use, it is more approachable to beginners, particularly for educational and DIY purposes. Kali Linux and Aircrack-ng serve as a comparison to evaluate performance.

### 3.2. Wi-Fi Hacking with Microcontrollers

Wi-Fi-capable microcontrollers offer unique possibilities for Wi-Fi security testing. They do not run Linux, but their firmware runs close to the hardware, enabling capabilities such as capturing and sending raw frames. These compact chips, designed for IoT products, can be used to build small, lightweight, inexpensive, and battery-powered security testing devices. Installing the firmware is straightforward with web flashers such as ESP Web Tools (https://esphome.github.io/esp-web-tools/, accessed on 15 May 2026), and further configuration is typically not required.

However, their system resources are limited. They are designed to perform simple tasks reliably with little memory and processing power. Using them instead of a general-purpose computer means foregoing the flexibility of a broad catalogue of Linux tools capable of running simultaneously or in conjunction. This trade-off is acceptable when the use case does not require complex or custom workflows, as such tools often complement rather than replace existing setups.

**Figure 5:**
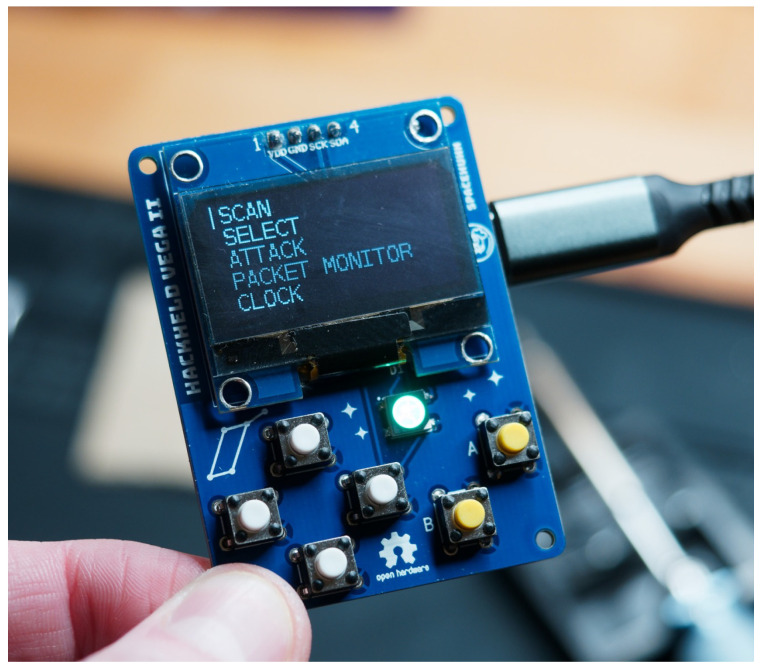
ESP8266 Deauther Handheld: “HackHeld Vega II”.

ESP8266 Deauther [1] (https://deauther.com/docs/about, accessed on 15 May 2026) is a popular and accessible tool for Wi-Fi deauthentication attacks and beacon spamming that we developed prior to this article. Based on the ESP8266 Wi-Fi microcontroller from Espressif, it is open-source and can be quickly built by flashing the firmware onto an inexpensive ESP8266 development board. With a small set of hardware buttons and a display, it can operate as a standalone handheld device without requiring a phone or computer to control it, as seen in Figure 5. Only the ESP8266 acts as the limiting factor. The small amount of memory, General-Purpose Input/Output (GPIO) pins, and older SDK make it hard to implement new features, which is why it is no longer maintained. This tool served as the predecessor and foundation for the prototypes developed in this article, establishing key goals such as affordability and DIY accessibility.

The ESP8266’s capabilities are further demonstrated in “Deauthentication of IP Drones and Cameras” [9], highlighting how this low-cost microcontroller can disconnect drones and cameras using Wi-Fi.

**Figure 6:**
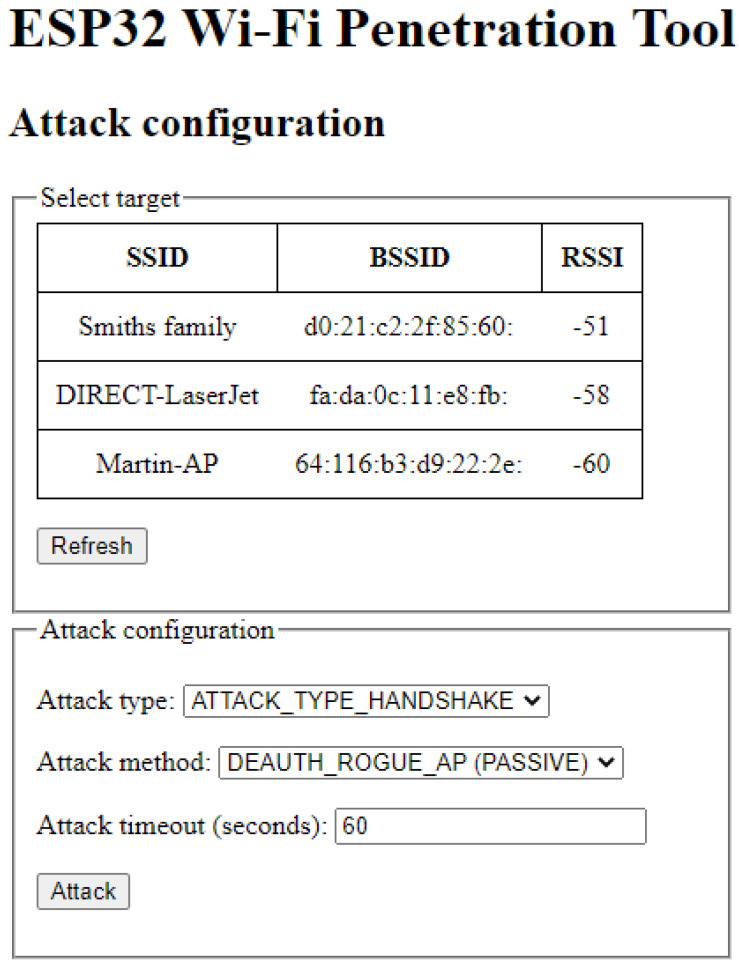
ESP32 Wi-Fi penetration tool web interface UI.

Other tools, such as the ESP32 Wi-Fi Penetration Tool [10], aim to replicate and improve upon the ESP8266 Deauther’s feature set. However, this tool provides only a rudimentary web-based UI, as shown in Figure 6 (image from the project’s README (https://github.com/risinek/esp32-wifi-penetration-tool, accessed on 15 May 2026)), and lacks standalone handheld capability. This tool is also examined in the paper “Wi-Fi Attacks Using ESP32” [11].

A further study [12] empirically analyses deauth attacks using the ESP8266 Deauther and Flipper Zero on IoT devices, evaluating impact and detection challenges. Unlike this article, the study focuses on attack effects rather than tool development.

The ESP32 represents a significant improvement over the ESP8266, and more capable variants are now available. Because the ESP32 Wi-Fi Penetration Tool already demonstrates that the ESP8266 Deauther’s functionality can be recreated on an ESP32, this article focuses on building a standalone handheld instead. Using the latest hardware and comparing performance against Linux-based tools serves to evaluate whether such a device can effectively replace professional tooling.

Slipper Zero [13] is an ESP32-based toolkit for deauth attacks, PMKID capture, and rogue APs to analyse wireless threats in real time. It emphasises low-cost offensive capabilities but lacks on-device app management, GUI-driven standalone operation, or comparative benchmarks against Kali tools.

### 3.3. Hacking with Microcontrollers Beyond Wi-Fi Applications

Beyond Wi-Fi, microcontrollers have been applied to a range of hardware security research, as demonstrated by the following publications:

ESPwn32—Hacking with ESP32 System-on-Chips [14] explores how the ESP32 can be exploited for attacks on BLE, Zigbee, and the ANT protocol. With techniques such as BLE fuzzing, jamming, and cross-protocol injections, this work highlights the potential of microcontrollers in wireless security research beyond Wi-Fi.

IRREM: Infrared Reverse Engineering Multitool [15] details the development of a tool designed to analyse and exploit vulnerabilities in infrared-controlled devices. Similar to this article, it uses a microcontroller as the hardware foundation, although it targets infrared rather than Wi-Fi.

Spyduino: Arduino as a HID Exploiting the BadUSB Vulnerability [16] introduces a tool leveraging an Arduino Uno to perform BadUSB attacks, mimicking a USB keyboard to execute commands on the target computer. This project illustrates the broader applicability of microcontroller platforms to security research.

“Threat Analysis of Portable Hack Tools from USB Storage Devices” [17] analyses portable hack tools on U3 USB platforms (e.g., portable apps for keylogging, privilege escalation) as threats to endpoint security, proposing detection via USB behaviour monitoring. Unlike our battery-powered handheld with on-device app management, it focuses on USB stick-based attacks without custom hardware or Wi-Fi capabilities.

“Challenges in Medical Device Communication: A Review of Security and Privacy Concerns in Bluetooth Low Energy (BLE)” [18] reviews BLE security in medical handheld devices, detailing attacks like jamming and eavesdropping and proposing privacy enhancements. This BLE-focused work contrasts with our 2.4 GHz Wi-Fi deauth/beacon tool, emphasising protocol analysis over portable attack implementation.

Hotplug-Attack-Tools – Überblick und Vergleich gängiger Tools [19] surveys hotplug attack tools, including the ESP8266 Deauther, providing context for the variety of existing hardware-based security tools and their use cases.

### 3.4. Differentiation from Existing ESP32 Tools

While the ESP32 Wi-Fi Penetration Tool demonstrates the feasibility of porting deauthentication functionality to the ESP32 platform, it addresses only one dimension of practical usability. The key limitations of existing ESP32-based tools are:

- Interface limitation: Existing tools rely on web-based or command-line interfaces, requiring a separate device (phone, laptop, or tablet) to operate, limiting practical deployment scenarios.
- Form factor: No existing ESP32 tool integrates hardware and firmware into a unified, truly standalone handheld device with native display and input controls.
- Accessibility: Installation and operation typically require setting up a development environment, which creates a barrier to entry for hobbyists and educational use.
- Hardware utilisation: Prior work does not fully exploit the ESP32-S3’s frame injection and capture capabilities, leaving performance and reliability gaps unaddressed.

This article directly addresses these gaps by developing the HackHeld32—a fully integrated, standalone handheld platform combining custom hardware with modern GUI-driven firmware. The custom bootloader and on-device app manager further differentiate this work, eliminating the need for a development computer. The comprehensive evaluation against professional tools validates the practical viability of this handheld-first approach.

### 3.5. Wi-Fi Hacking with Single-Board Computers

In addition to using regular computers or microcontrollers, a Single-Board Computer (SBC) like the Raspberry Pi (https://www.raspberrypi.com, accessed on 15 May 2026) provides the full potential of Linux in a small form factor.

P4wnP1 A.L.O.A. (https://github.com/RoganDawes/P4wnP1_aloa, accessed on 15 May 2026) is a project that demonstrates the capabilities of Raspberry Pi-based solutions for hacking. Using the Raspberry Pi Zero W, this multi-purpose Pentesting framework supports Wi-Fi, Bluetooth, and BadUSB attacks.

“Discovering Public Wi-Fi Vulnerabilities Using Raspberry Pi and Kali Linux” [20] demonstrates how a Raspberry Pi can perform Wi-Fi attacks like DNS spoofing, password cracking, MiTM, and Evil Twin. However, their portable setup, costing around 100 €, relies on multiple external components, making it less compact and cost-effective than the fully integrated, standalone approach explored in this article.

“Defense and Analysis of Hijacking User Login Credentials via Remote Code Execution and Raspberry Pi” [21] explores another use case of the Raspberry Pi for physical access attacks. The authors demonstrate how a Raspberry Pi can be used to implant malware that mimics a Windows login screen, capturing login credentials and sending them to a remote server.

When performance requirements exceed microcontroller capabilities, a Single-Board Computers (SBCs) provides a practical alternative with full Linux tooling in a compact form factor. The trade-off is higher system complexity and cost because OS setup, storage, and peripheral requirements are more demanding than for microcontroller-based builds.

The Clockwork uConsole (https://www.clockworkpi.com/uconsole, accessed on 15 May 2026) is an open-source modular handheld built around the Raspberry Pi Compute Module. It offers a polished portable Linux platform but sits in a higher price class than typical DIY microcontroller builds.

**Figure 7:**
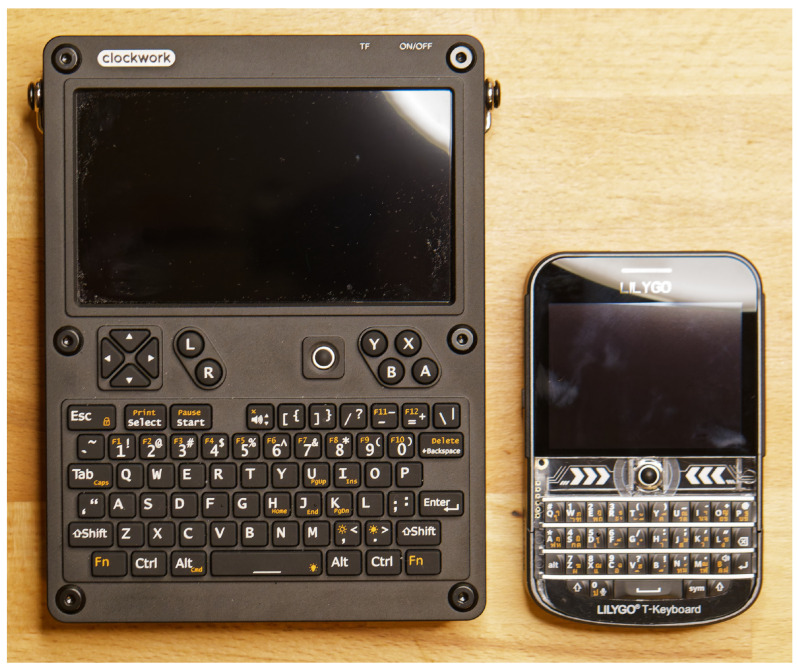
Clockwork uConsole and LILYGO T-Deck Plus.

Meanwhile, a ready-to-use and feature-rich ESP32-S3-based handheld such as the LILYGO T-Deck Plus (https://lilygo.cc/products/t-deck-plus, accessed on 15 May 2026) is available for 68 €. Figure 7 shows both devices, with the uConsole on the left and the T-Deck on the right.

Considering setup complexity, portability constraints, and cost, this article adopts a microcontroller-based approach to prioritise affordability, accessibility, and standalone operation. SBCs remain appropriate when full Linux flexibility is required.

## 4. Hardware Development

**Figure 8:**
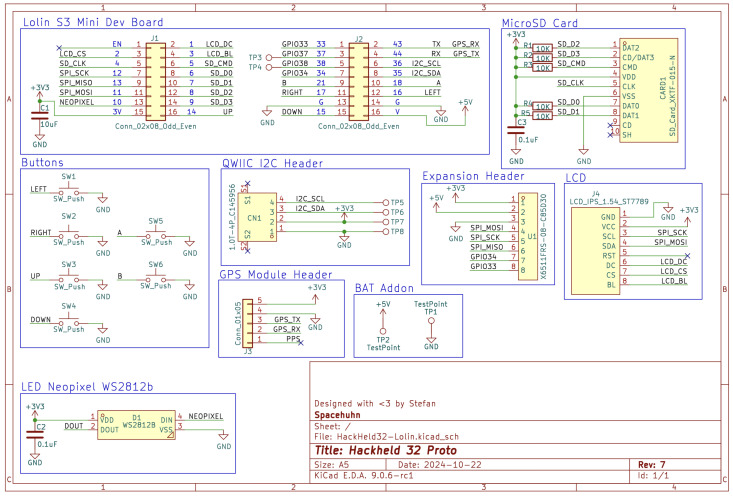
HackHeld32 PCB Schematic.

This project builds on the proven design of the HackHeld Vega. The working title, HackHeld32, nods to its predecessor while highlighting the upgrade to the ESP32-S3. Espressif’s Wi-Fi-enabled microcontrollers are the preferred choice, offering a large community, excellent documentation, and a wide variety of affordable development boards. Their support for open-source libraries and platforms like Arduino sets them apart from alternatives such as the Realtek Ameba (https://www.amebaiot.com/en/control-mcu, accessed on 15 May 2026) series. The ESP32-S3 was selected for its dual-core CPU and 45 GPIO pins, well-suited for a standalone handheld. Its dual-core architecture allows Wi-Fi-related tasks to run independently from other operations, preventing interference with the Wi-Fi stack. It remains inexpensive compared to SBC alternatives while offering sufficient performance for the intended use case. The ESP32-C5 would be an exciting choice due to its 5 GHz Wi-Fi capabilities. Unfortunately, it was unavailable for purchase at the time of writing. To make development quicker, the HackHeld32 Printed Circuit Board (PCB) keeps the same 4.5 cm width and 6 cm height as the HackHeld Vega. Some components, like buttons and LEDs, also share the same position as the predecessor. KiCad (https://www.kicad.org, accessed on 15 May 2026), a free and open-source electronics design suite, was used to create the hardware design. Figure 8 shows the schematic, detailing the connections between components.

### 4.1. Parts and Components for the HackHeld32

**Figure 9:**
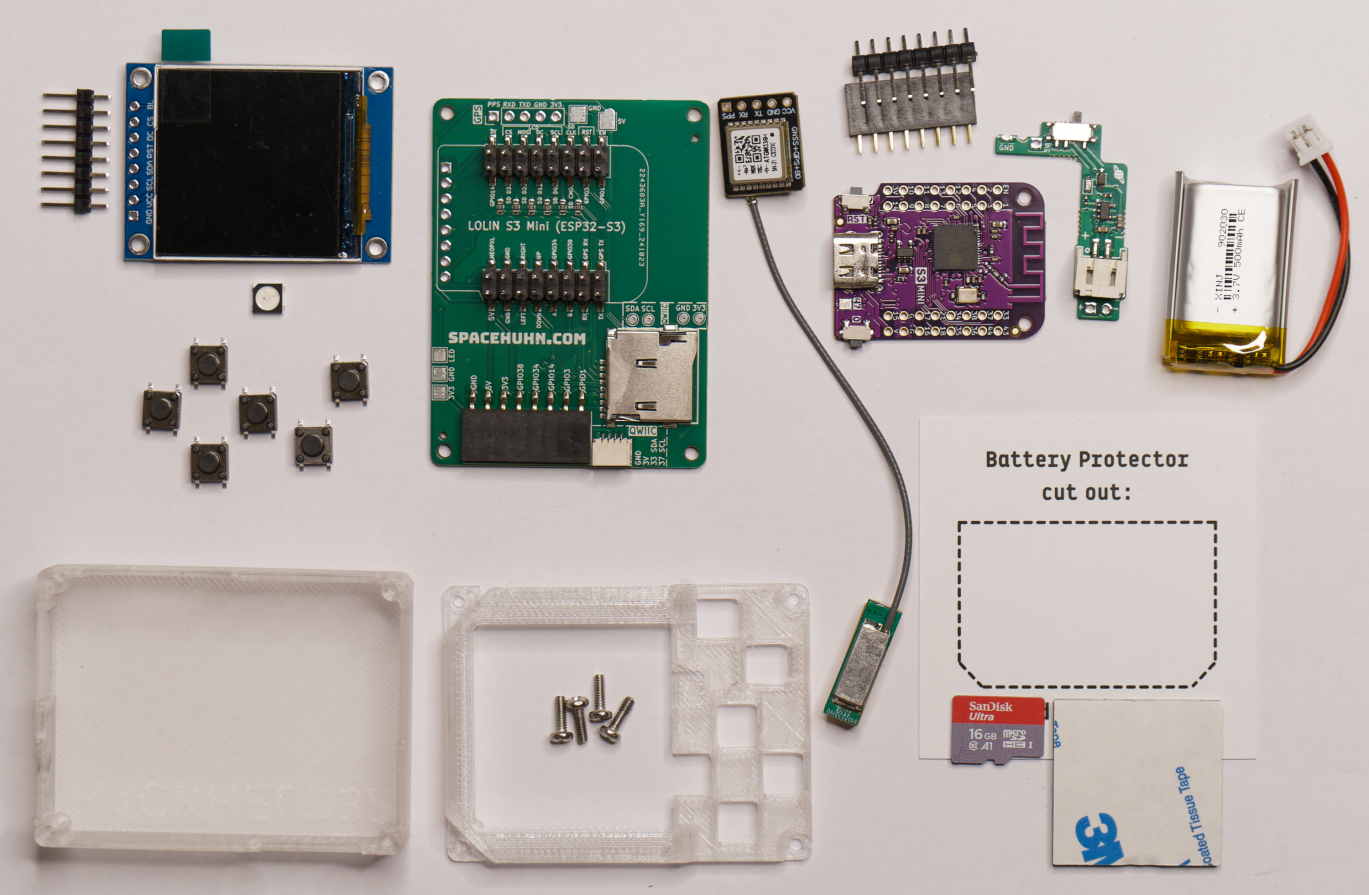
Parts for HackHeld32.

Table 2 lists the selected parts for building the HackHeld32, also visible in Figure 9. Prices are taken from AliExpress (https://aliexpress.com, accessed on 15 May 2026), except for the PCB, charging module, and case, which were calculated using quotes from JLCPCB (https://jlcpcb.com, accessed on 15 May 2026) by uploading the design. The total for all parts is 35.09 €.

Due to the design approach, it is possible to omit components such as the GPS module or battery, which can still be added later, thereby lowering the overall cost. Table 3 provides an overview of the essential parts required to assemble a functional HackHeld32 while maintaining its core functionality. This dramatically cuts down the cost to just 15.60 €, which is about the same price as the parts for the HackHeld Vega.

All parts are selected to be affordable, easy to find and easy to assemble. Only the surface-mounted components for the microSD card slot require soldering experience. Buttons, LEDs, and pin headers are ordinary and can be replaced with similar, compatible parts. Other parts are more crucial and, therefore, discussed in their subsections below. The Lolin S3 Mini was selected as the development board because it matches the footprint of the Lolin D1 Mini used on the HackHeld Vega and provides sufficient GPIO pins for all components; larger ESP32-S3 boards do not fit the PCB. The display is a 1.54-inch 240 × 240 colour LCD, which governs the device’s physical dimensions and significantly upgrades the 128 × 128 monochrome OLED of the predecessor while keeping the overall footprint unchanged. The 3D-printed case reuses the HackHeld Vega’s dimensions, requiring only small cutouts for the microSD slot. An optional GPS module (ATGM336H) connects via a pin header, enabling future wardriving applications.

### 4.2. HackHeld32 PCB Design

**Figure 10:**
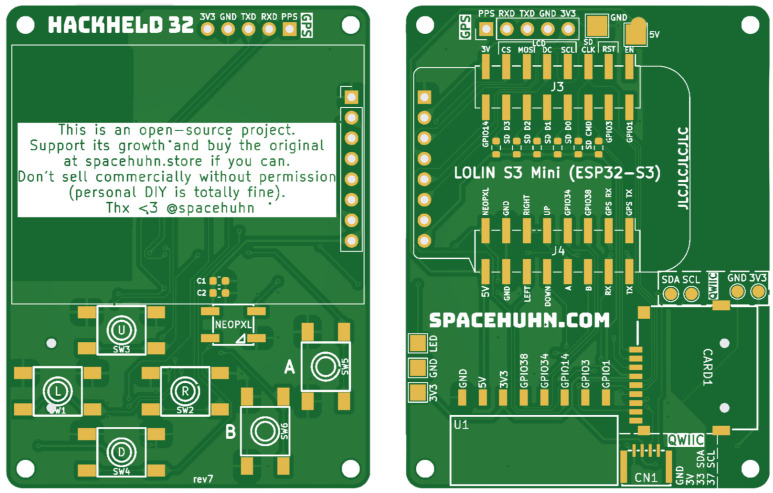
HackHeld32 PCB Design (rev7).

The PCB design underwent seven revisions. The first revision established core connectivity but contained wiring errors and a defective LiPo battery circuit. Subsequent revisions corrected these faults, offloaded battery management to an external module, added a GPS pin header, exposed unused GPIO as breakout pads, and replaced copper-pad expansion points with a 2.54 mm pin header relocated to the bottom edge for ergonomic add-on support. Later revisions introduced a Qwiic (https://www.sparkfun.com/qwiic, accessed on 15 May 2026) connector for standardised plug-and-play module support and added copper pads for in-case debugging. The final design (rev7), shown in Figure 10, is the version used throughout this work.

### 4.3. HackHeld32 Case Design

**Figure 11:**
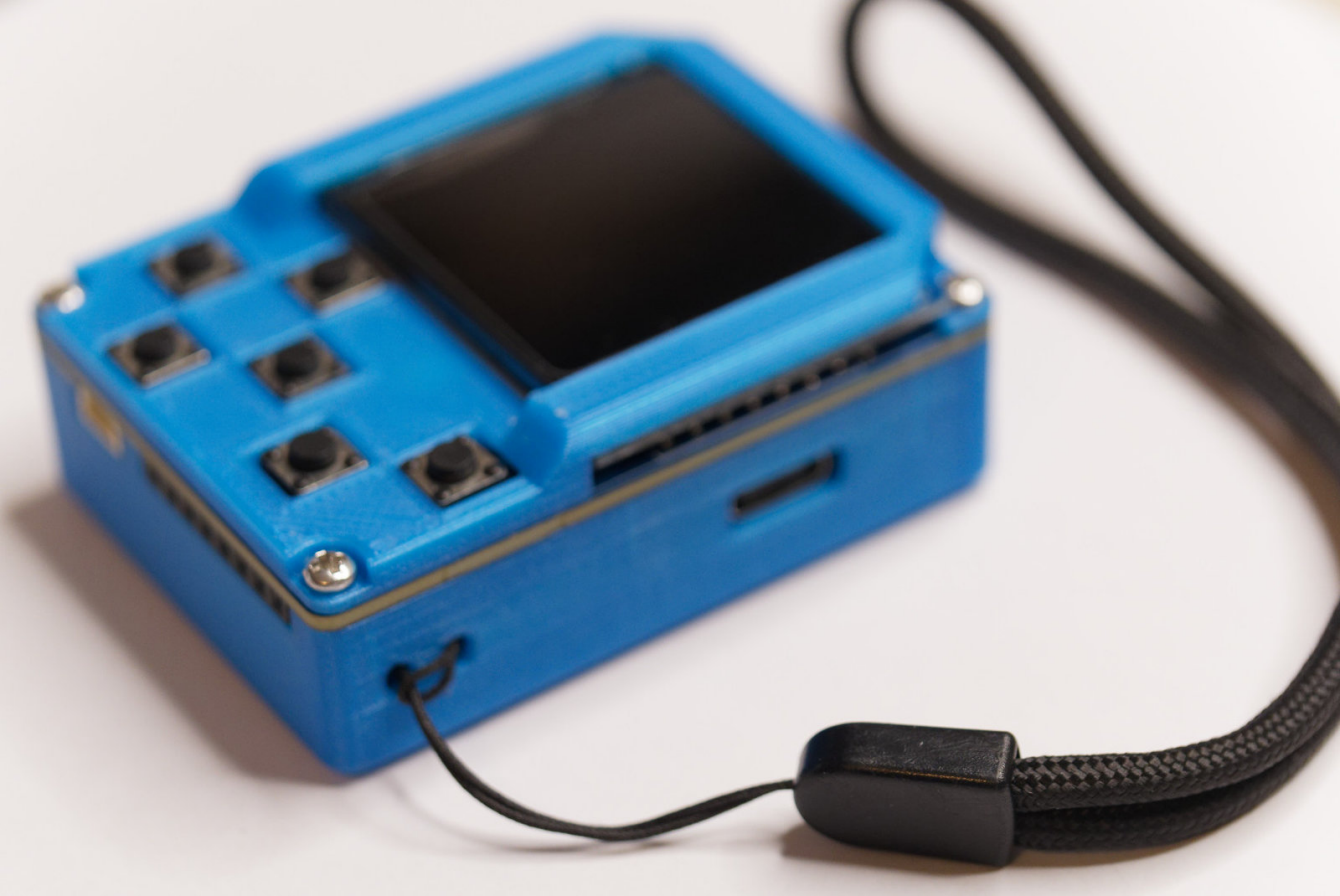
HackHeld32 with Case and Wristband.

The case was designed using OpenSCAD 2021.01 (https://openscad.org, accessed on 15 May 2026), a free and open-source 3D CAD modelling software. The plastic enclosure for the device underwent many iterations. Given the iterative nature of 3D design and printing, most changes are minor and focus primarily on testing and ensuring proper fit and alignment. Like other parts of this design, the back cover was also based on the HackHeld Vega. Since the PCB is the same size, adapting the existing case design for new components, like the microSD card slot, made sense. The front cover, however, needed to be designed from the ground up due to the different screen. The completed device with its case and wristband is shown in Figure 11.

### 4.4. Resulting Hardware

**Figure 12:**
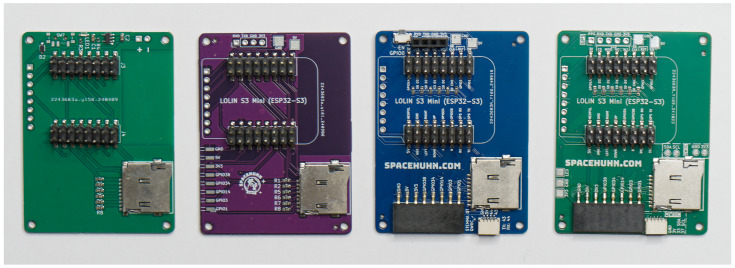
HackHeld32 PCB revisions (left to right: rev1, rev3, rev6, rev7).

**Figure 13:**
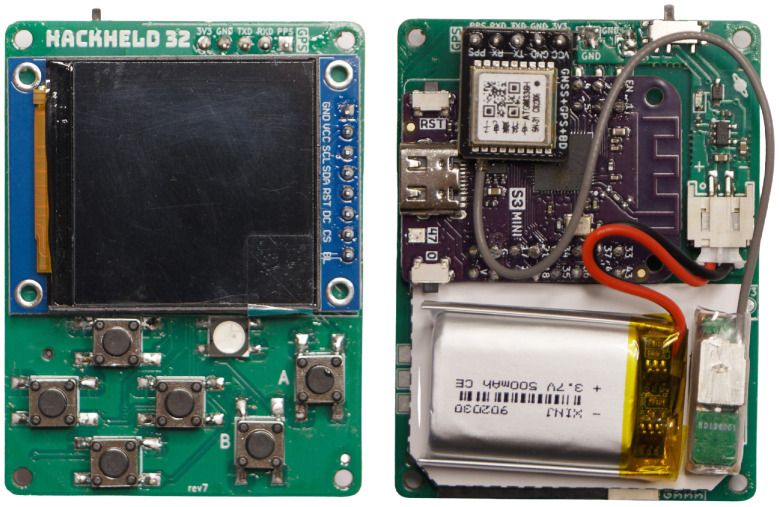
HackHeld32 PCB rev7 (front and back).

**Figure 14:**
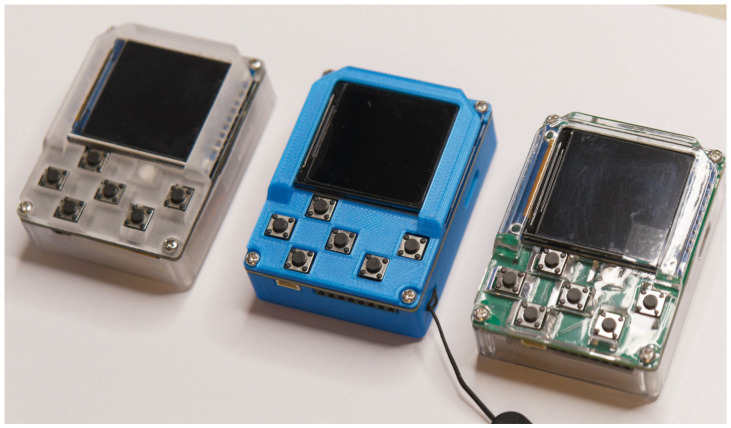
HackHeld32s with different coloured cases.

**Figure 15:**
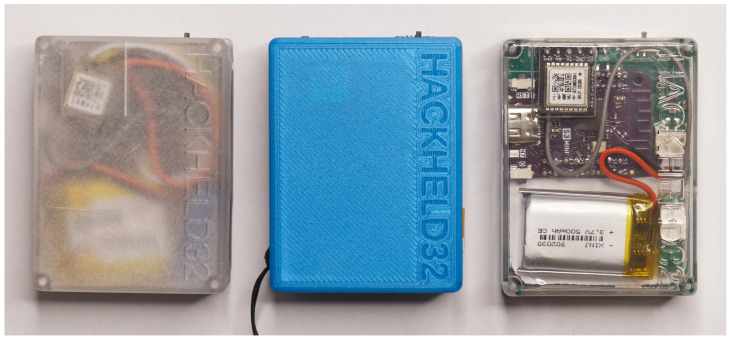
HackHeld32s with different coloured cases (back view).

**Figure 16:**
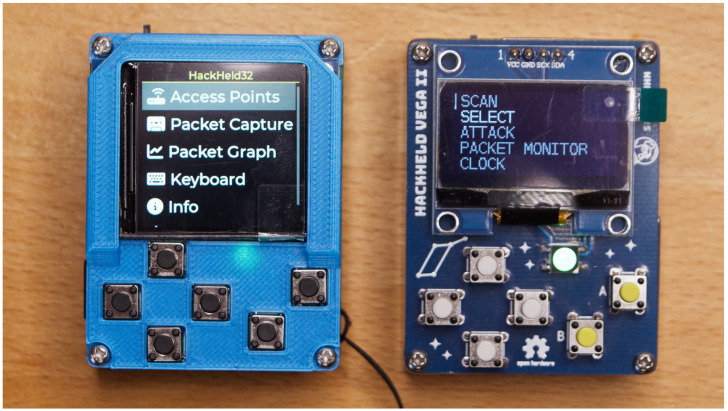
HackHeld32 (A) and HackHeld Vega (B) side by side.

Figure 12, Figure 13, Figure 14, Figure 15 and Figure 16 show the PCB revision history, the assembled rev7 board, and the completed device alongside its predecessor.

## 5. Firmware Development

The basis for firmware development is the **Espressif IoT Development Framework (ESP-IDF)** (https://docs.espressif.com/projects/esp-idf/en/latest/esp32, accessed on 15 May 2026). It provides all the necessary libraries and tools for developing, compiling, and flashing applications onto the ESP32, including the ESP32-S3 used in this project. It is written in C for its efficiency and close-to-hardware nature. However, the ESP-IDF also provides C++ support, allowing the use of modern and higher-level features. **Arduino** (https://www.arduino.cc, accessed on 15 May 2026) was selected to simplify development and modifications. Arduino is an open-source platform that provides standardised libraries and tools to abstract much of the hardware complexity. This allows developers to focus on functionality rather than low-level implementation details. Arduino’s simplicity and widespread adoption make the project more accessible for users to contribute and modify code. The Arduino core for the ESP32 is built on top of the ESP-IDF, maintaining full access to the underlying interfaces. Arduino’s extensive library ecosystem and active community also contribute to faster development and integration of different components.

The following Arduino libraries were used for this project:

- TaskScheduler (https://github.com/arkhipenko/TaskScheduler, accessed on 15 May 2026) for managing recurring tasks.
- LVGL (https://github.com/lvgl/lvgl, accessed on 15 May 2026) for creating the GUI.
- Adafruit_GFX (https://github.com/adafruit/Adafruit-GFX-Library, accessed on 15 May 2026) and **Adafruit_ST7789** (https://github.com/adafruit/Adafruit-ST7735-Library, accessed on 15 May 2026) for interfacing with the screen.
- Adafruit_NeoPixel (https://github.com/adafruit/Adafruit_NeoPixel, accessed on 15 May 2026) for controlling LEDs.
- TinyGPSPlus (https://github.com/mikalhart/TinyGPSPlus, accessed on 15 May 2026) for parsing GPS data.
- ArduinoJson (https://github.com/bblanchon/ArduinoJson, accessed on 15 May 2026) for working with JSON data.

### 5.1. Graphical User Interface (GUI)

**Figure 17:**
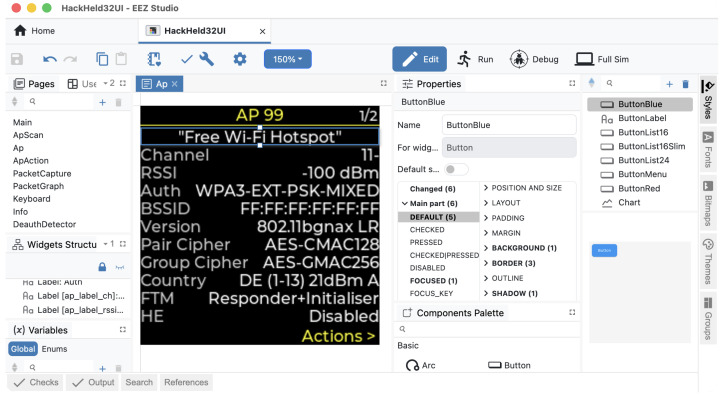
EEZStudio.

**Figure 18:**
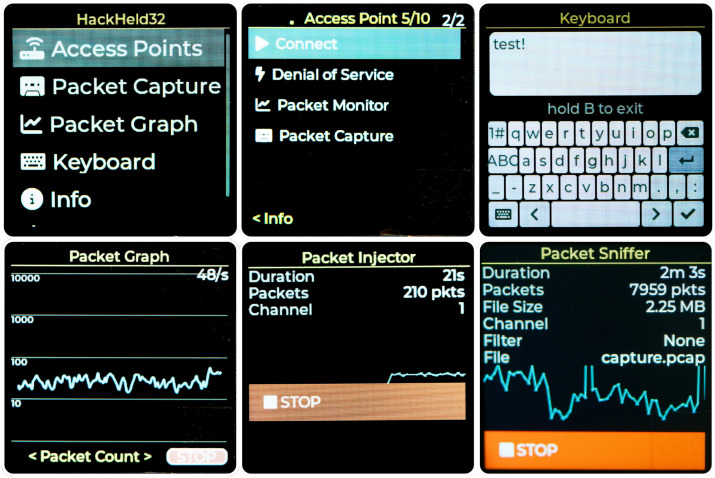
HackHeld32 GUI Pages.

The Adafruit_ST7789 library, used to interface with the screen, allows drawing shapes, text and images. Unfortunately, it does not provide functionality for building a UI. Another problem that arose was the noticeably slow full-screen redraw. To make the UI more responsive, it is necessary to keep track of changes and only redraw parts of the screen that have changed. The **LVGL library** solved these problems. It is a powerful and flexible library for building GUIs for embedded devices, handling navigation and partial screen redraws. Its Arduino support makes it easy to integrate into the codebase and use with the ESP32-S3. **EEZStudio** (Figure 17) helped build the GUI visually, significantly speeding up the design process. It is a free and open-source visual GUI editor designed for embedded devices and LVGL. The result is a responsive interface that is intuitive and easy to use. Figure 18 displays some of the pages built for the HackHeld32 prototype using this approach.

### 5.2. Bootloader and App Manager

Throughout the development, it became evident that writing a single firmware that unites various Wi-Fi hacking features is very time-consuming. Some features are easier to develop and test as a dedicated application. For example, capturing Wi-Fi traffic is resource-intensive and requires careful optimisation for the best results. A large codebase with many features is more challenging to maintain and debug.

However, having multiple single applications conflicts with the goal of creating a device that is easy to use. If the user had to manually reflash the firmware with a computer whenever they wanted to use a different feature, it could no longer be considered a standalone device.

Throughout this article, “application” (app) and “firmware” are used interchangeably, with “app” referring to firmware for the HackHeld32 prototype and “firmware” as a broader term.

#### 5.2.1. Partition Table and Bootloader

The ESP-IDF uses a partition table (https://docs.espressif.com/projects/esp-idf/en/stable/esp32/api-guides/partition-tables.html, accessed on 15 May 2026), which allows the storage of multiple applications in the flash memory. Besides the primary app, additional slots can be added to enable over-the-air (OTA) updates. This presented an opportunity to make a system where users can switch between the installed apps.

The ideal solution would resemble a dual-boot setup on a computer, where the user selects the desired app after starting the device. To achieve this, a custom bootloader was necessary. Otherwise, the ESP32-S3 would always boot into the primary app. Creating a fully custom bootloader is not a trivial task. With a memory limitation of 4 KB, it is practically impossible to implement a GUI.

**Figure 19:**
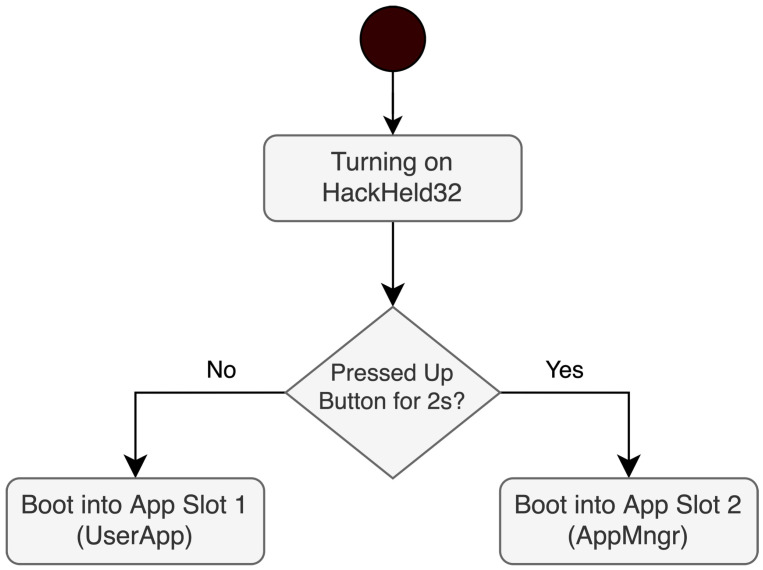
Bootloader App Selection Flow.

**Figure 20:**
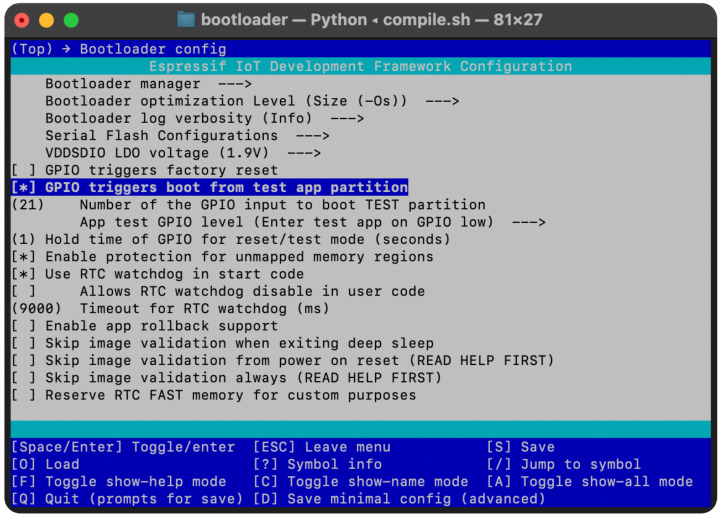
ESP-IDF menuconfig displaying the option to boot from the second app slot.

To keep things as simple as possible, the default bootloader was used as a basis and reconfigured to listen to a button press at boot time. If the up button on the HackHeld32 is pressed for two seconds when the device starts, it will boot into the app stored in the second app slot. Otherwise, it will start the app stored in the first slot. Figure 19 visualises this process. This feature is configurable via the menuconfig tool in the ESP-IDF as seen in Figure 20. Table 4 shows the customised partition table with UserApp being the first and AppMngr being the second app.

#### 5.2.2. App Manager

**Figure 21:**
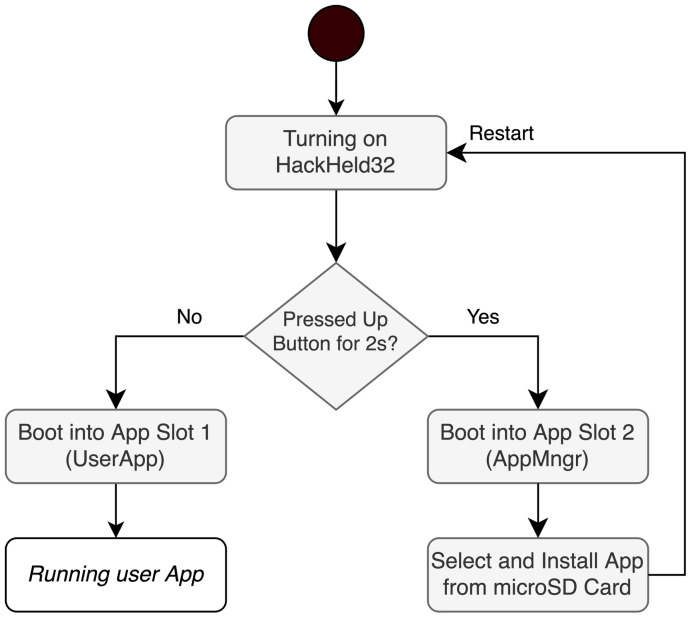
App Installation Flow.

**Figure 22:**
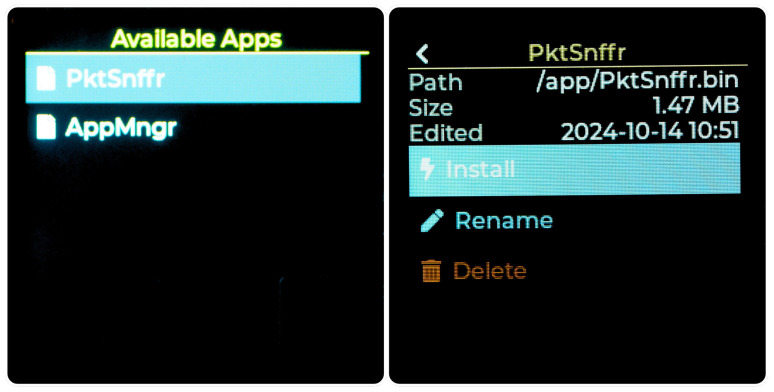
App Manager GUI.

Because the bootloader only allows users to select between two applications, one of them has to make it possible to select, install, and start other applications. Therefore, the App Manager (AppMngr) was created. It is a standard firmware capable of utilising the full range of hardware and software features. But it is stored in the second app slot, which is only booted into if the configured button is pressed at boot time. It is marked with the subtype test in the partition table to ensure that, after a reboot, the bootloader defaults back to the first app slot for launching the user application. In its current implementation, the App Manager scans the connected microSD card for .bin files and lists them on screen. The user can select one of the files and install it. To add a new app to the selection, the user has to copy the app’s .bin file onto the SD card. After installation, the device restarts and boots into the newly installed application. Figure 21 visualises the boot process. The App Manager’s GUI can be seen in Figure 22. This approach leads to the primary application being completely independent and unaware of the App Manager in the second slot, making it possible for users to write custom applications for the device without the need to include any special code for this functionality. Whether the primary app is working or not, it is always possible to start the App Manager. Because the user application is stored at the default address, flashing a different firmware onto the device through Arduino will not override the App Manager.

## 6. Wi-Fi Sniffer Implementation

This section describes the implementation of a Wi-Fi sniffer on the ESP32-S3 and evaluates its performance against a Linux laptop and a Wi-Fi Pineapple. The results determine whether the ESP32-S3 offers a meaningful alternative for capturing Wi-Fi traffic and identify the hardware constraints that bound its applicability.

### 6.1. PktSnffr App Implementation

The Packet Sniffer (PktSnffr) app for the HackHeld32 captures Wi-Fi traffic and saves it on the microSD card as a .pcap file. Software like Wireshark 4.6.5 (https://www.wireshark.org, accessed on 15 May 2026) can be used to view and analyse the Wi-Fi traffic.

Considering that the ESP32-S3 is intended for IoT applications that do not require high data throughput, optimising the way the captured traffic is saved is essential to make this app work as intended.

For this reason, the microSD card is connected through the ESP32-S3’s SDMMC interface (https://docs.espressif.com/projects/esp-idf/en/v5.3.1/esp32s3/api-reference/peripherals/sdmmc_host.html, accessed on 15 May 2026) to achieve the best throughput. Leveraging the ESP32-S3’s ability to operate this interface at 40 MHz using 4-bit communication supports theoretical transfer rates of up to 20 MB/s (160 Mbit/s). For comparison, using the 2.4 GHz band with a single antenna results in a theoretical maximum Wi-Fi throughput of 150 Mbit/s, assuming optimal conditions.

Some code optimisation was necessary to utilise the high-speed SDMMC interface fully. A double-buffering mechanism with two 64 KB buffers ensures that if one buffer is full, it can be written to the microSD card while incoming traffic gets saved into the second buffer. Direct Memory Access (DMA) reduces CPU overhead by handling large chunks of data directly between memory and the microSD card. This approach yields the best possible throughput when the microSD card is formatted to FAT32 with a 64 KB allocation unit size.

An Arduino sketch was created to perform a speed test to determine the most capable microSD card for this project. The test involves writing a 64 KB buffer to the card once, ten times, and a hundred times to evaluate the card’s performance across varying write capacities. All cards were formatted to FAT32 with a 64 KB allocation unit size. The results are presented in Table 5. The Kioxia card demonstrated the highest write speeds and was therefore selected for use in this project.

### 6.2. PktSnffr GUI

**Figure 23:**
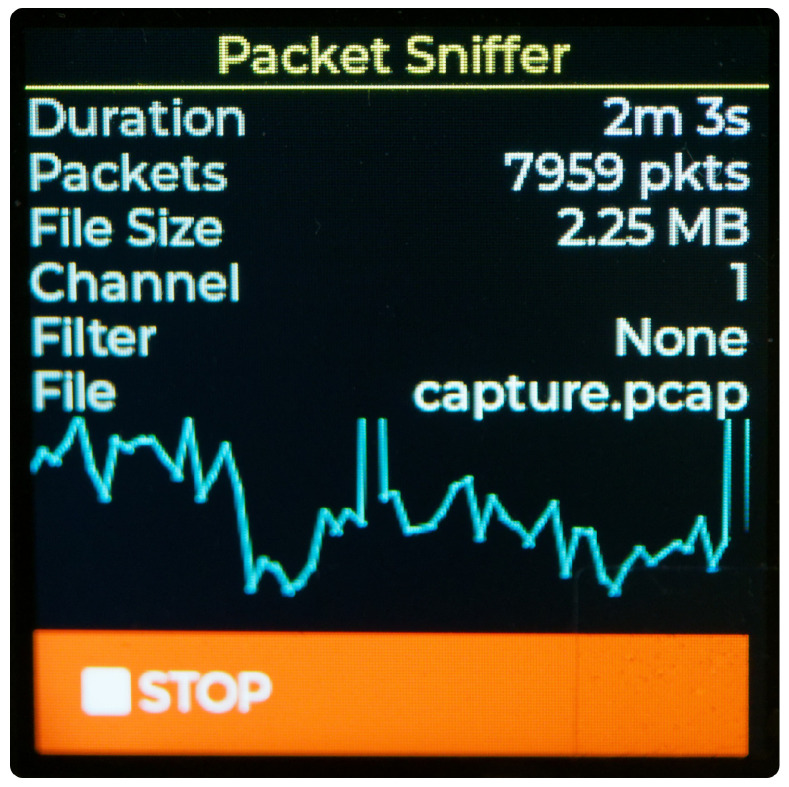
Packet Sniffer App GUI.

The GUI, as shown in Figure 23, is a simple single-page view that shows the runtime, number of packets or frames, file size, selected channel, selected filter and the active capture, as well as a small graph visualising the packets per second. Starting and stopping the capture is possible using the button at the bottom of the screen.

By utilising both the ESP-IDF filter capabilities and custom checks in the promiscuous callback function, it is possible to reduce memory usage and processing overhead. This feature is hardcoded at the time of writing, but it is planned to provide several filter options, and the GUI already features an element for selecting them.

### 6.3. Capture Performance Evaluation

The HackHeld32 was evaluated against a Framework 13-inch (11th-gen Intel) laptop running Kali Linux with an RTL8188 USB Wi-Fi adapter, and in selected tests against a Hak5 Wi-Fi Pineapple equipped with large external antennas. All devices listened simultaneously on channel 6 of the 2.4 GHz band; the HackHeld32 saved frames to a microSD card while the laptop used Wireshark. Each test lasted 60 s, with conditions (placement, orientation, and obstacles) held constant within each test series. Placement and antenna orientation were observed to affect results, particularly in range tests; no repeated runs were performed.

#### 6.3.1. Low-Traffic Performance (Tests 1–6)

**Figure 24:**
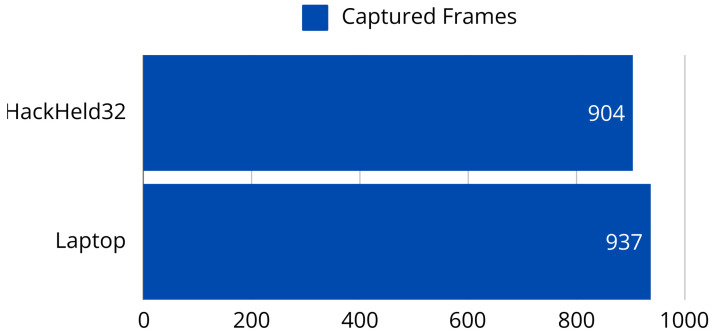
Capture Test 1 Frame Count.

**Figure 25:**
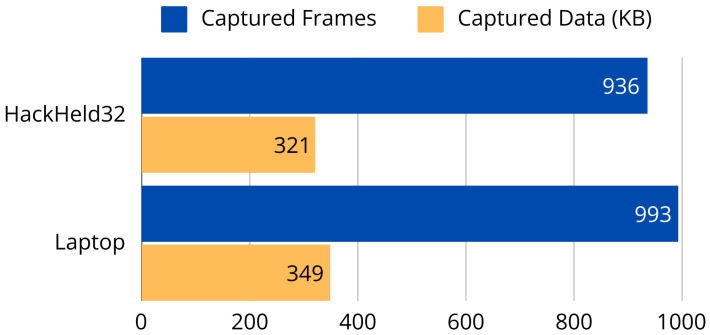
Capture Test 2 Frame Count and Size.

Tests 1 through 6 were conducted with fewer than five idle devices on a single network. In Test 1, both devices counted received frames without saving them. The HackHeld32 received 904 frames versus 937 on the laptop (Figure 24), confirming comparable passive reception. When saving was enabled in Test 2, the HackHeld32 captured 936 frames (321 KB) against 993 frames (349 KB) on the laptop (Figure 25); the additional frames carried weak Received Signal Strength Indicator (RSSI) values (−85 to −91 dBm), indicating a minor sensitivity disadvantage rather than a throughput issue.

**Figure 26:**
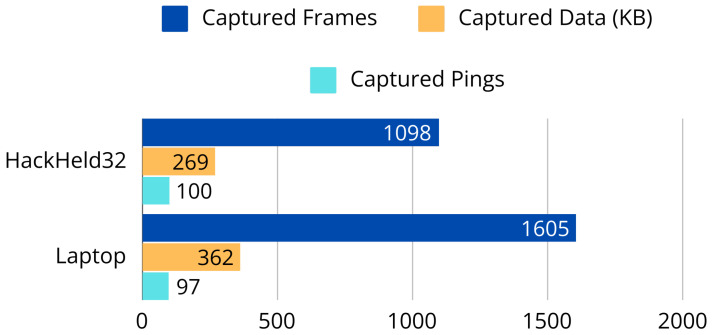
Capture Test 3 Frame and Ping Count.

**Figure 27:**
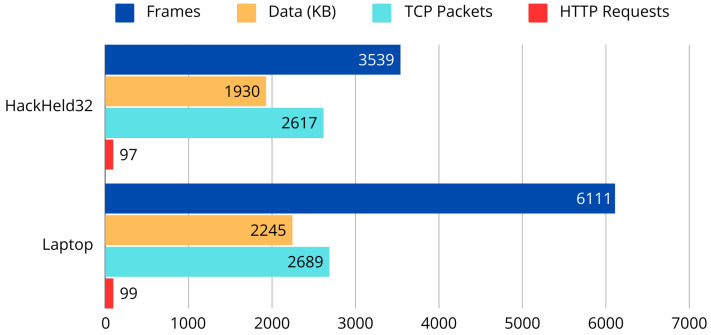
Capture Test 4 Frame and HTTP Count.

**Figure 28:**
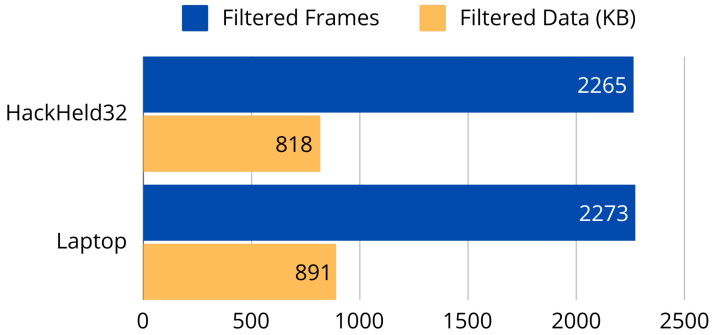
Capture Test 5 Filtered Frame Count and Size.

**Figure 29:**
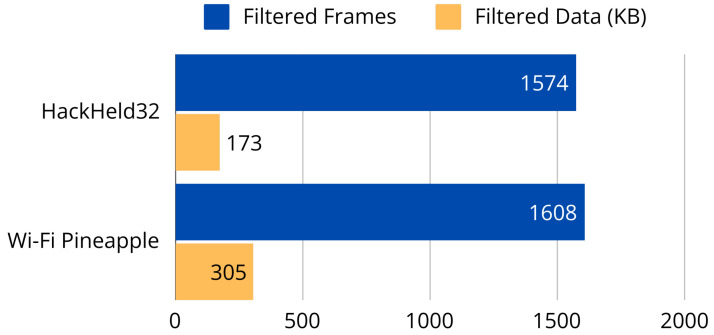
Capture Test 6 Filtered Frame Count and Size.

Tests 3 and 4 introduced controlled traffic from a single device. For 100 ICMP pings (Test 3), the HackHeld32 captured all 100 packets while the laptop captured 97, despite the laptop recording more total frames (Figure 26). For 100 HTTP GET requests to google.com (Test 4), the HackHeld32 recorded 2617 TCP packets, including 97 HTTP requests, versus 2689 TCP and 99 HTTP on the laptop (Figure 27); the larger total frame count difference (3539 vs. 6111) reflects background traffic rather than missed target packets. Applying a MAC-address filter in Test 5 brought both devices to near-identical performance at 2265 frames (818 KB) versus 2273 frames (891 KB) (Figure 28), confirming that on-device filtering closes the throughput gap for targeted capture. Test 6 substituted the Wi-Fi Pineapple for the laptop; the Pineapple captured 2608 frames (305 KB) against 1574 frames (173 KB) on the HackHeld32 (Figure 29), a gap attributable to the Pineapple’s external antennas rather than processing capability.

#### 6.3.2. High-Traffic Performance (Tests 7–8)

**Figure 30:**
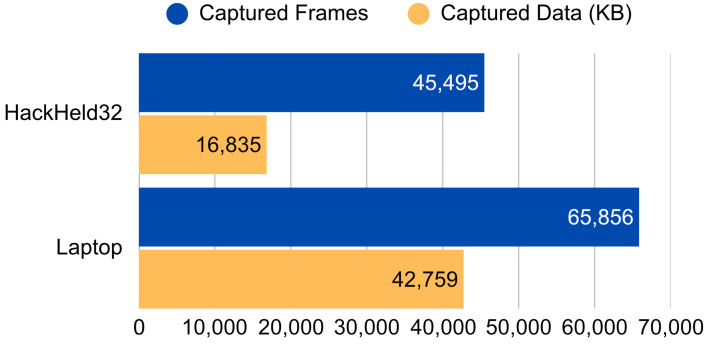
Capture Test 7 Frame Count and Size.

**Figure 31:**
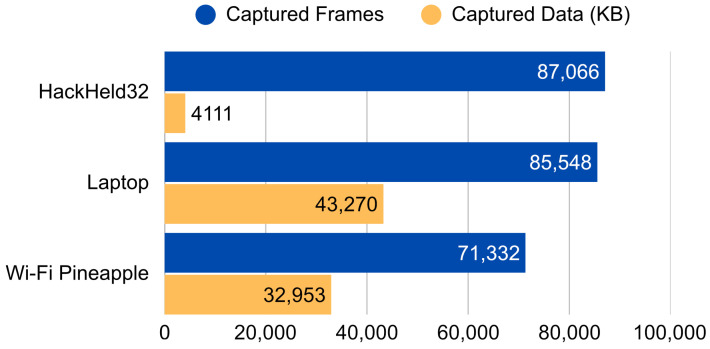
Capture Test 8 Frame Count and Size.

Tests 7 and 8 were conducted with a device streaming high-resolution video over a dedicated 2.4 GHz AP, with no capture filter applied. In Test 7 with full frame capture, the HackHeld32 recorded 45,495 frames (16,835 KB) against the laptop’s 65,856 frames (42,759 KB) (Figure 30), revealing a clear throughput bottleneck: large-payload frames were dropped once the write buffers were exhausted, while small-payload frames were handled reliably. Test 8 addressed this by saving only the MAC header and discarding the frame payload, which is typically encrypted and of limited analytical value. Under the same high-traffic conditions, the HackHeld32 recorded 87,066 frames (4111 KB), exceeding both the laptop’s 85,548 frames and the Wi-Fi Pineapple’s 71,332 frames in frame count (Figure 31). Header-only mode fully mitigates the throughput limitation at the cost of payload data.

#### 6.3.3. Range Evaluation (Tests 9–10)

**Figure 32:**
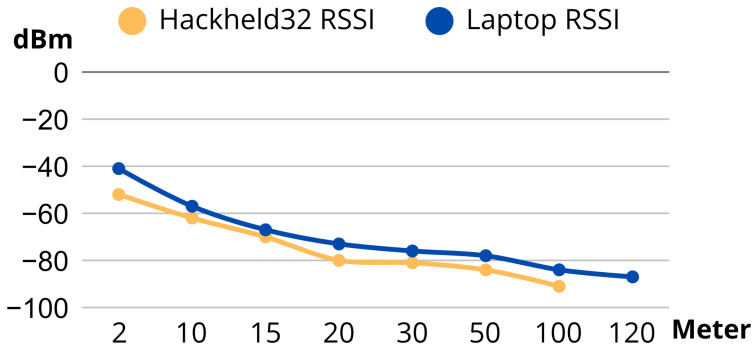
Capture Test 9 Outdoor Range Test.

**Figure 33:**
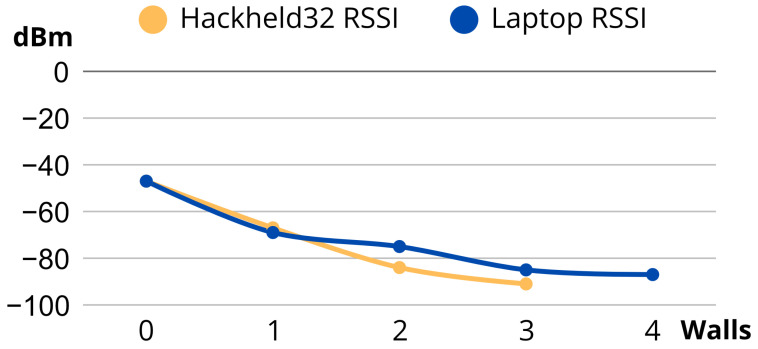
Capture Test 10 Indoor Range Test.

Test 9 measured RSSI from a single AP’s beacon frames at increasing distances in an open outdoor area with no nearby interference. The laptop maintained reliable reception to approximately 120 m; the HackHeld32 lost reliable signal at around 100 m (Figure 32). Physical handling had a measurable effect: fully gripping the device attenuated the antenna, while a two-finger hold improved reception. Test 10 repeated the evaluation indoors, using wall count as the primary metric (distance remained within 15 m). Within one wall of the AP, both devices performed similarly. Beyond two walls, the HackHeld32 degraded noticeably, losing reliable reception at four walls versus five for the laptop (Figure 33). The results confirm that the HackHeld32 is best suited to same-room or adjacent-room deployments; the observed range shortfall is consistent with the compact PCB antenna’s relative size compared to the laptop’s larger Wi-Fi module.

### 6.4. Limitations and Future Work

The HackHeld32’s primary hardware constraint is throughput: in high-traffic environments, large-payload frames are dropped once the write buffers are exhausted. Header-only capture mitigates this at the cost of payload data, which is typically encrypted and of limited forensic value. The compact PCB antenna reduces reception range by approximately 20 m outdoors and by one wall indoors relative to a laptop-class Wi-Fi interface; this is inherent to the ESP32-S3 module and cannot be addressed at the firmware level. The evaluation methodology relied on single 60-s runs without repetition, limiting statistical confidence, particularly in range tests where placement effects were clearly present.

Future development priorities include configurable filter presets and user-defined filter profiles, which would improve usability in targeted capture scenarios. Enriching the .pcap output with RSSI, channel, and precise timestamp metadata—or adopting the pcapng format—would improve compatibility with modern analysis workflows. Integrating a Wi-Fi AP and station scanner would allow users to identify and select target devices before capture, reducing reliance on prior knowledge of target MAC addresses.

## 7. Packet Injection Implementation

Packet injection allows transmitting custom-defined Wi-Fi frames generated outside the Wi-Fi interface’s internal network stack. These frames can imitate (spoof) legitimate network traffic from other devices, enabling manipulation such as forcing disconnections or advertising nonexistent networks. The following sections implement and evaluate beacon flooding and deauthentication as two representative attacks on the ESP32-S3, assessing HackHeld32’s suitability as a Wi-Fi security testing tool.

### 7.1. ESP-IDF Constraints and Workaround

The ESP-IDF used to build firmware for the ESP32 family includes the esp_wifi_80211_tx() function to allow sending custom Wi-Fi frames. Unfortunately, it does not permit unrestricted packet injection: it only supports beacon, probe request, probe response, action, and non-QoS data frames, silently rejecting all others. The source code responsible for this behaviour is hidden in the ESP-IDF’s core libraries, which are only supplied as compiled binaries, making direct modification difficult. Weakening the ieee80211_raw_frame_sanity_check symbol via objcopy did not bypass the restriction in practice. Direct library patching with a disassembler, demonstrated by Project Zonde [22] for older ESP-IDF versions, is fragile because compiled binary structure changes between ESP-IDF releases, obsoleting patch offsets. The selected approach is patching the compiled firmware image at the app level. The build process generates a .elf file that retains symbol information, allowing the frame-validation logic to be located and overridden before conversion to a flashable .bin image. The ESP32-deauther project (https://github.com/GANESH-ICMC/esp32-deauther, accessed on 15 May 2026) uses this strategy, and its patch.sh workflow served as the baseline for the HackHeld32 implementation. This method avoids rebuilding vendor libraries, supports a reproducible patching pipeline, and keeps the overridden function in user-controlled source code; ESP-IDF version pinning ensures the patch remains stable across builds.

### 7.2. Beacon Flood Implementation

**Figure 34:**
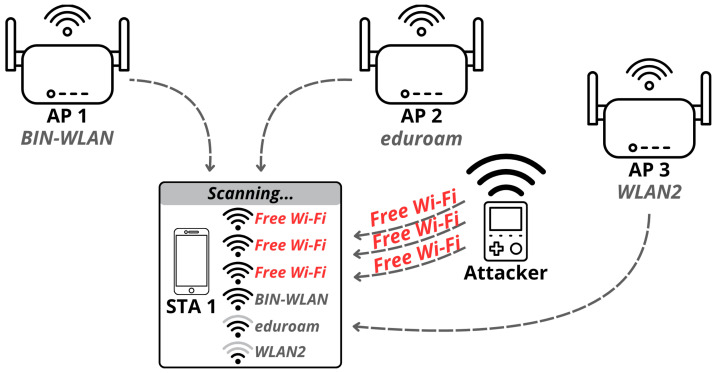
Wi-Fi Beacon Flood.

A beacon frame flood sends many fabricated beacon frames, each advertising a non-existent Wi-Fi network with an arbitrary SSID. These frames populate the Wi-Fi scan lists of nearby devices with fake networks, as visualised in Figure 34. For security testing, this technique is valuable for evaluating how devices and users respond to SSID spoofing and for testing detection and filtering mechanisms.

**Figure 35:**
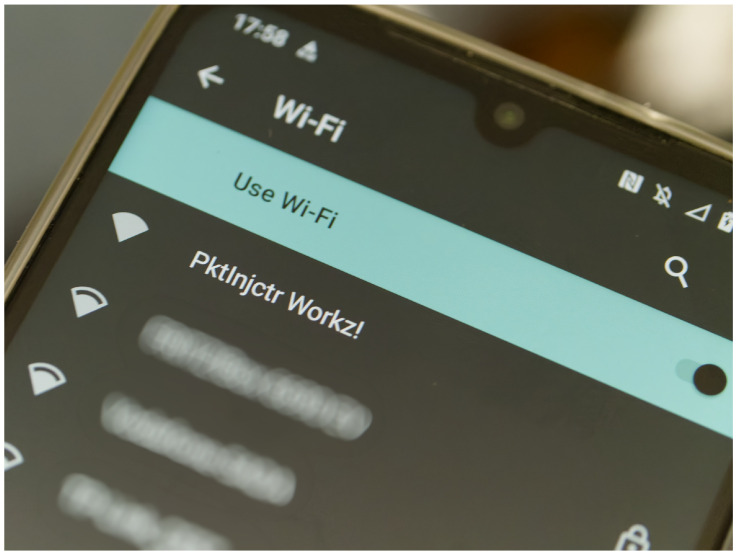
Beacon Frame Injection Result: “PktInjctr Workz!”.

The packet injector app (PktInjctr) uses esp_wifi_80211_tx() and a fixed beacon frame definition, with the patch script applied after compiling as described above. Within less than a minute of starting the app, nearby Wi-Fi devices detect the advertised SSID, as confirmed in Figure 35, even though no physical AP provides that network.

### 7.3. Deauthentication Attack Implementation

**Figure 36:**
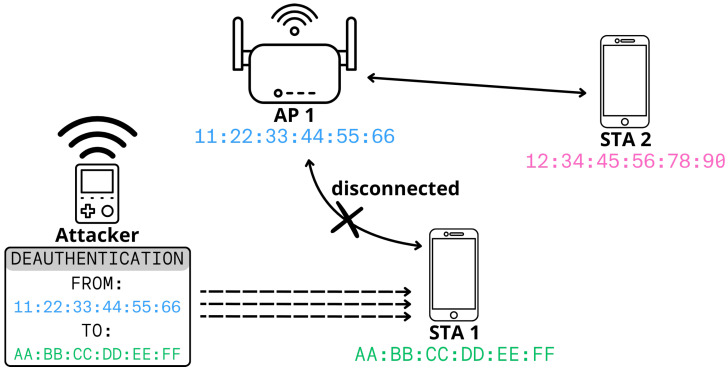
Wi-Fi Deauthentication Attack.

A Wi-Fi deauthentication (deauth) attack is a DoS attack that exploits spoofed deauthentication frames to force a device to disconnect from its network. An attacker sends deauth frames carrying the spoofed MAC addresses of the target AP and STA, as shown in Figure 36, repeatedly preventing reconnection. Frames can be sent as unicast (targeting a specific AP-ST pair) or broadcast (targeting all clients of a given AP), depending on the attacker’s objective.

The implementation follows the same approach as the beacon flood, with only the frame definition changed, as shown in Listing 1. The app successfully deauthenticated the selected target.

**Listing 1.** Deauthentication Frame Structure.
data ={

/*  0 - 1  */ 0xC0, 0x00,  // type, subtype c0: deauth (a0: disassociate)

/*  2 - 3  */ 0x00, 0x00,  // duration (SDK takes care of that)

/*  4 - 9  */ 0xFF, 0xFF, 0xFF, 0xFF, 0xFF, 0xFF, // receiver (target)

/*  10 - 15  */ 0x00, 0xE0, 0x4B, 0x9B, 0xC8, 0x98, // source (ap)

/*  16 - 21  */ 0x00, 0xE0, 0x4B, 0x9B, 0xC8, 0x98, // BSSID (ap)

/*  22 - 23  */ 0x00, 0x00,  // fragment & sequence number

/*  24 - 25  */ 0x01, 0x00   // reason code (1 = unspecified reason)

}


### 7.4. Evaluating Sender Range

Two range tests evaluated packet injection effectiveness by comparing the RSSI of beacon frames sent by the HackHeld32 against those from a reference Wi-Fi router, measured with airodump-ng on a Kali Linux laptop.

**Figure 37:**
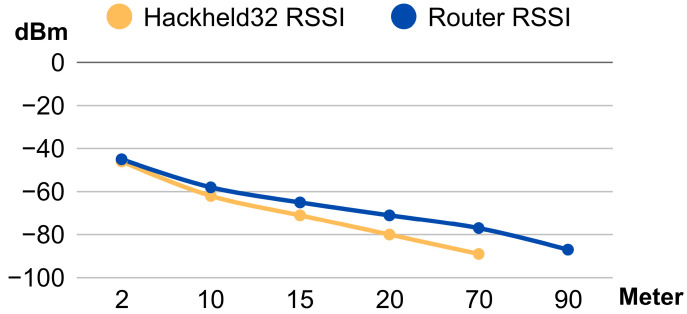
Packet injection outdoor range test.

The outdoor test was conducted without obstacles and without nearby active Wi-Fi devices. Both devices were placed on the ground at a fixed position, and RSSI values were recorded at increasing distances. As shown in Figure 37, values were similar within 10 m, but the HackHeld32’s signal dropped significantly at greater distances. At around 70 m, its frames became unreliable, compared to approximately 90 m for the reference router.

**Figure 38:**
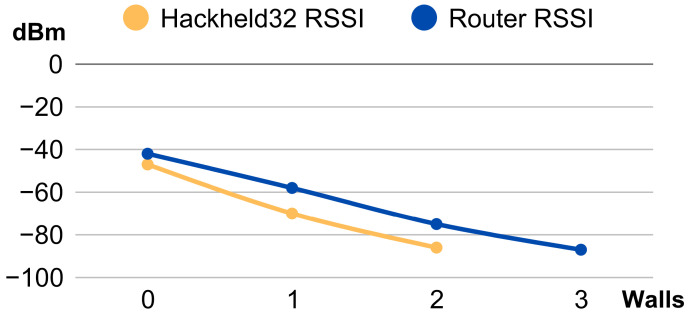
Packet injection indoor range test.

The indoor test was conducted in a house with multiple nearby APs and active Wi-Fi devices, using the number of obstructing walls as the primary metric. Signal strength remained comparable within the same room. With one wall, the router already showed a noticeably stronger signal. After two walls, the HackHeld32’s frames were barely receivable, versus three walls for the reference router, as shown in Figure 38.

Outdoors, frames maintain a reliable signal up to 10–15 m and reach a maximum of approximately 70 m. Indoors, performance is best with no more than one obstructing wall. The HackHeld32’s weaker antenna and lower transmission power, attributable to the ESP32-S3’s energy-efficient design, limit its effective range compared to a dedicated router. This is an acceptable trade-off for a compact, cost-effective tool intended for short-range security testing.

## 8. Deauther32 Prototype Development

With the working HackHeld32 prototype and its ability to capture and send raw Wi-Fi frames, the next logical progression is to combine these capabilities into a firmware that can succeed the ESP8266 Deauther [1]. The primary objective for a “Deauther32” is to match the feature set of the ESP8266 Deauther, covering AP and STA scanning, network traffic visualisation, and deauthentication and beacon flood attacks. Extending the feature set beyond the ESP8266 Deauther is of secondary importance, as Deauther32 must first be able to replicate its predecessor.

### 8.1. Main Menu

**Figure 39:**
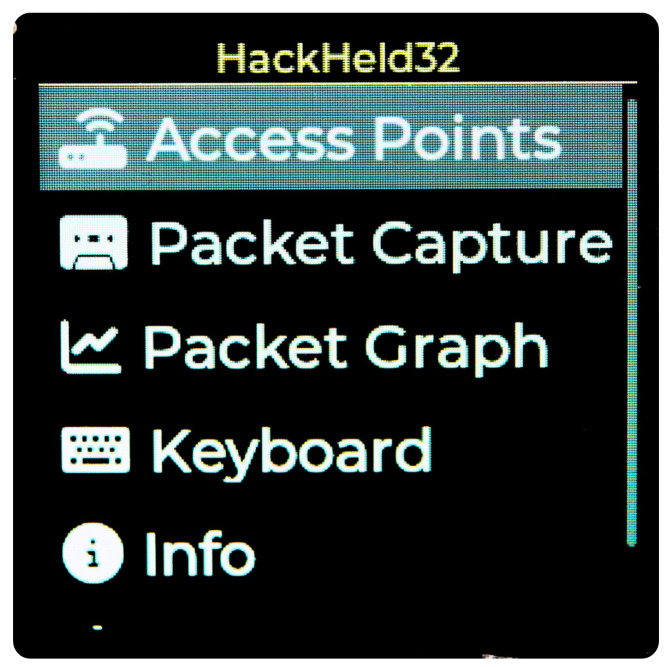
Deauther32 main menu page.

The main menu of Deauther32, as seen in Figure 39, is a minimalistic scrollable list with the names and matching icons of the respective functions for quick navigation. The Up and Down buttons are used to scroll through the list, and the A button is used to open the selected page.

### 8.2. AP Scanning

**Figure 40:**
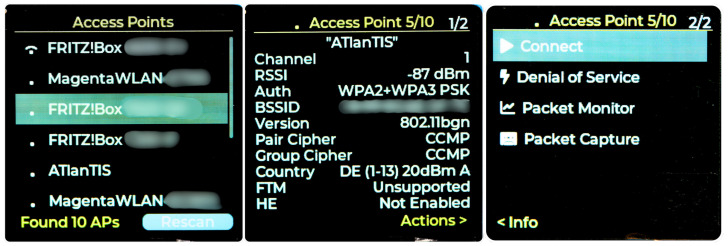
Deauther32 AP scanner and detail page.

The AP Scanning page scans and lists nearby APs. Selecting an AP opens a detail page that displays channel, RSSI, authentication type, and BSSID. Pressing the right button opens an action page with controls for packet capture and DoS attacks; in the current prototype, these controls remain placeholders. Figure 40 shows the AP scanner and list on the left, with the AP detail page next to it and the action page on the right.

### 8.3. Packet Capture and Packet Graph

**Figure 41:**
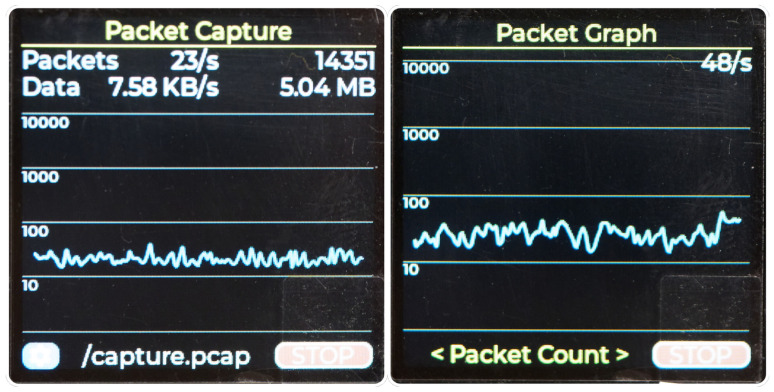
Deauther32 packet capture and graph page.

The Packet Capture page allows users to capture Wi-Fi traffic, draw a graph of the received frames per second, and display metrics such as packet count and data rate. Starting and stopping the capture is done by clicking the button next to the file name at the bottom. The Packet Graph is a simplified version of the same page without the capture metrics, allowing a larger graph. It only visualises the network traffic without capturing it. Both pages use a logarithmic graph to display frame rates, making it easier to interpret large variations over time and visualise both low and high-traffic scenarios in one view. Figure 41 depicts the capture page on the left and the graph page on the right.

## 9. Conclusions and Outlook

**Figure 42:**
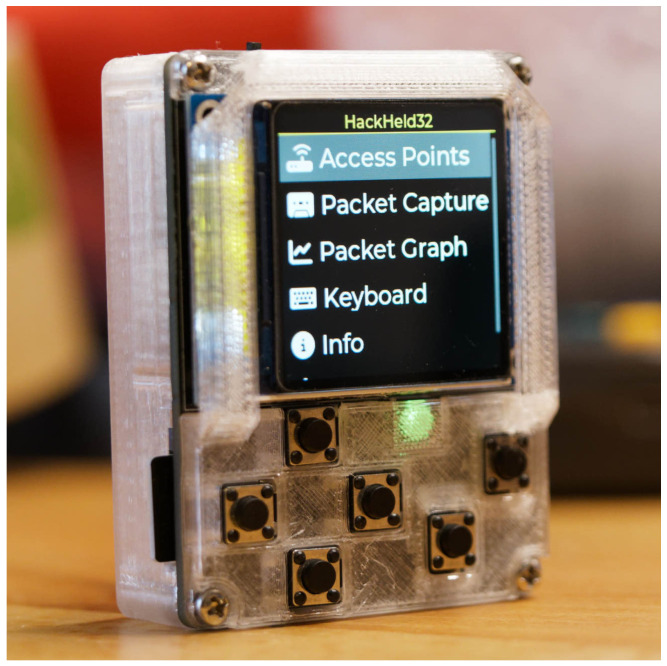
Assembled HackHeld32 running Deauther32.

The prototypes we developed, tested, and documented in this article (Figure 42) demonstrate the ESP32-S3’s capabilities and the feasibility of creating a low-cost, portable Wi-Fi hacking tool. By enabling frame capture and injection, the project highlights the platform’s potential, with tests confirming that the signal range remains sufficient for targeting nearby devices.

Building on our earlier projects, the ESP8266 Deauther [1] and HackHeld Vega [23], this project addresses their limitations by using the newer and more capable ESP32-S3 microcontroller instead of the now-outdated ESP8266. The proposed Deauther32 firmware prototype showcases promising potential as a successor to the ESP8266 Deauther. It provides several improvements, such as leveraging the ESP32-S3 and ESP-IDF capabilities and introducing a modern GUI. However, it still needs to be further developed to match the feature set of its predecessor.

The designed HackHeld32 hardware (Section 4) complements this firmware as a compact DIY-friendly handheld. Compared to the HackHeld Vega, its upgrades include a full-colour LCD screen, an optional GPS module, microSD card support, and support for connecting modules or sensors, enabling hardware extensions. The device remains affordable at around 35 € in full configuration and 15 € in minimal configuration.

By integrating a custom bootloader and App Manager (Section 5.2), users can switch between applications without reflashing the microcontroller with a computer, allowing for the development of additional apps and extending functionality beyond Wi-Fi hacking.

Future hardware work could still explore a lower-cost ESP32-C3 variant, antenna or case refinements, or a preassembled non-DIY version for users who prefer greater convenience over modifiability.

Although more work is needed to realise the full potential of Deauther32 and HackHeld32, the project meets key goals (Section 1.1) such as ease of use, portability, affordability, modifiability, a small form factor, battery-powered operation, and support for basic Wi-Fi hacking features like frame capture and injection. Overall, the article establishes a strong foundation for future work, demonstrating the practicality of the ESP32-S3 for Wi-Fi hacking tools.

### 9.1. Use Cases and Applications

The Deauther32 and HackHeld32 prototypes enable Wi-Fi hacking tasks like frame capture, injection, and testing attacks such as beacon floods and deauthentication. These capabilities make the devices useful for network security assessments, educational purposes, and real-world testing.

The ESP32-S3-based project is particularly appealing to beginners, students, and hobbyists due to its affordability, compactness, and ease of use, making it an excellent entry point into Wi-Fi security. While test results reveal limitations in range and throughput—making it unsuitable for large traffic volumes—the DIY nature makes it ideal for demonstrations, testing new attack methods, portable field assessments, and as a complementary tool for professional setups.

Additionally, the modular hardware and firmware, combined with the affordability of the ESP32-S3, make these devices well-suited for teaching, prototyping wireless technologies, and creating custom tools. Through open-source collaboration and community engagement, this tool can evolve to support diverse use cases such as portable network monitoring and custom security tooling.

### 9.2. Outlook

While the presented work established the capabilities of the ESP32-S3, there are many opportunities for improvement and expansion. More advanced Wi-Fi attack and testing features could expand the device’s capabilities, enabling functionalities such as creating fake APs for phishing, performing MiTM attacks, and capturing four-way handshakes for offline password cracking. As the ESP32 cannot simultaneously operate as a rogue AP (to trigger reconnects) and packet sniffer due to single-radio constraints, adding a second Wi-Fi interface would allow for Evil Twin attacks.

The Deauther32’s usability could be improved by adding other interface methods, such as a web-based or mobile application, alongside the screen GUI. Automation features, including predefined attack presets and logging options, would make the tool more accessible for less experienced users. Additionally, achieving feature parity with the ESP8266 Deauther is a key milestone to ensure the Deauther32 is a complete replacement.

The App Manager system and hardware expandability invite users to create and integrate custom features to suit their needs. Improving the antenna could address some of the range limitations observed during testing, and exploring alternative ESP32 variants might make the project more versatile. Expanding firmware compatibility to additional hardware, such as the LILYGO T-Deck, and adapting GUI elements for various screen resolutions and input methods would allow it to be used across different devices.

Finally, advanced testing in controlled environments with simulated interference, diverse network configurations, and stress-testing scenarios would provide valuable insights into the device’s limitations and optimisation possibilities.

## Figures and Tables

**Table 1:** OSI Model Layers in Wi-Fi.

	Layer	Description
7	Application	Data
6	Presentation	Data
5	Session	Data
4	Transport	Segments
3	Network	Packets
2	Data Link	Frames (802.11 MAC)
1	Physical	Bits (802.11 PHY)

**Table 2:** HackHeld32 Parts List.

Part	Quantity	Price (Total)
1.54″LCD Screen	1	3.05 €
Lolin S3 Mini Dev. Board	1	7.09 €
GPS Module	1	4.79 €
LED	1	0.09 €
Buttons	6	0.35 €
PCB	1	4.15 €
Case (back + front)	2	1.32 €
Screws	4	0.04 €
Battery charging module	1	3.00 €
LiPo Battery	1	7.12 €
MicroSD Card	1	4.09 €

**Table 3:** HackHeld32 Minimal Parts List.

Part	Quantity	Price (Total)
1.54″ LCD Screen	1	3.05 €
Lolin S3 Mini Dev. Board	1	7.09 €
Buttons	6	0.35 €
PCB	1	4.15 €
Case (back)	1	0.92 €
Screws	4	0.04 €

**Table 4:** ESP 32 Partition Table.

Name	Type	SubType	Offset	Size
nvs	data	nvs	0x9000	0x5000
otadata	data	ota	0xe000	0x2000
UserApp	app	ota_0	0x10000	0x1E0000
AppMngr	app	test	0x1F0000	0x1E0000
spiffs	data	spiffs	0x3D0000	0x20000
coredump	data	coredump	0x3F0000	0x10000

**Table 5:** MicroSD Card Write Performance Benchmarks.

Card	64 KB	640 KB	6400 KB
8 GB Class 4	1.40 MB/s	8.11 MB/s	9.06 MB/s
16 GB Class 4	1.11 MB/s	5.43 MB/s	2.90 MB/s
16 GB Class 10	4.04 MB/s	8.29 MB/s	9.15 MB/s
Amazon Basics 128 GB V30 U3 A2	8.86 MB/s	12.45 MB/s	12.71 MB/s
Kioxia 64 GB U1	9.11 MB/s	14.86 MB/s	15.26 MB/s
Lexar 633x 128 GB V30 U3 A1	8.89 MB/s	11.87 MB/s	12.07 MB/s
Paradise 8 GB Class 4	2.17 MB/s	4.26 MB/s	3.05 MB/s
Samsung EVO 64 GB U1	5.53 MB/s	8.40 MB/s	7.93 MB/s
Samsung EVO Plus 64 GB U3	6.27 MB/s	10.46 MB/s	10.56 MB/s
Samsung EVO Select 128 GB V30 U3 A2	2.60 MB/s	7.23 MB/s	8.09 MB/s
Sandisk 16 GB Class 10 U1	0.34 MB/s	2.84 MB/s	11.02 MB/s
Sandisk 32 GB Class 10 U1	6.74 MB/s	8.98 MB/s	9.38 MB/s
Sandisk Extreme 32 GB V30 U3 A1	3.03 MB/s	4.97 MB/s	7.50 MB/s
Sandisk Extreme 64 GB V30 U3 A2	6.25 MB/s	11.95 MB/s	12.10 MB/s
Sandisk Extreme 128 GB V30 U3 A2	4.42 MB/s	9.45 MB/s	10.32 MB/s
Sandisk Extreme Pro 32 GB V30 U3 A1	6.13 MB/s	10.55 MB/s	12.00 MB/s
Sandisk Extreme Pro 64 GB V30 U3 A2	3.63 MB/s	8.57 MB/s	9.62 MB/s
Sandisk High Endurance 32 GB V30 U3	4.37 MB/s	7.15 MB/s	8.26 MB/s
Sandisk High Endurance 64 GB V30 U3	3.62 MB/s	8.54 MB/s	9.10 MB/s
Sandisk Ultra 16 GB Class 10 A1	3.71 MB/s	4.32 MB/s	3.27 MB/s
Sandisk Ultra 32 GB Class 10 A1 U1	1.81 MB/s	8.27 MB/s	8.98 MB/s
Toshiba 4GB Class 4	0.16 MB/s	1.33 MB/s	4.50 MB/s
Transcend 16 GB Class 2	0.78 MB/s	7.60 MB/s	10.06 MB/s
Transcend Premium 400× 8 GB U1	3.46 MB/s	7.03 MB/s	7.12 MB/s
Verbatim Pro 32 GB Class 10 V30 U3	6.69 MB/s	12.61 MB/s	13.20 MB/s

## Data Availability

The original contributions presented in this study are included in the article. Further inquiries can be directed to the corresponding authors.

## Abbreviations

The following abbreviations are used in this manuscript:

AP	Access Point
BSSID	Basic Service Set Identifier
DIY	Do-It-Yourself
DoS	Denial-of-Service
ESP-IDF	Espressif IoT Development Framework
GPIO	General-Purpose Input/Output
GUI	Graphical User Interface
MAC	Medium Access Control
MiTM	Man-in-the-Middle
PCB	Printed Circuit Board
RSSI	Received Signal Strength Indicator
SBC	Single-Board Computer
SSID	Service Set Identifier
STA	Station
UI	User Interface
